# Phenotypic and genotypic characterization of single circulating tumor cells in the follow‐up of high‐grade serous ovarian cancer

**DOI:** 10.1002/1878-0261.70193

**Published:** 2025-12-23

**Authors:** Carolin Salmon, Rui P. L. Neves, Nikolas H. Stoecklein, Sven‐Thorsten Liffers, Jens Siveke, Jan D. Kuhlmann, Pauline Wimberger, Paul Buderath, Rainer Kimmig, Sabine Kasimir‐Bauer

**Affiliations:** ^1^ Department of Gynecology and Obstetrics University Hospital Essen Germany; ^2^ Department of General, Visceral and Pediatric Surgery University Hospital and Medical Faculty of the Heinrich‐Heine University Düsseldorf Germany; ^3^ West German Cancer Center, Bridge Institute of Experimental Tumor Therapy University Medicine Essen Germany; ^4^ Division of Solid Tumor Translational Oncology, German Cancer Research Center (DKFZ) and German Cancer Consortium (DKTK) Partner Site Essen 69120 Heidelberg Germany; ^5^ Department of Gynecology and Obstetrics, Medical Faculty and University Hospital Carl Gustav Carus Technische Universität Dresden Dresden Germany; ^6^ German Cancer Consortium (DKTK) Partner Site Dresden and German Cancer Research Center (DKFZ) Heidelberg Germany; ^7^ National Center for Tumor Diseases (NCT) Dresden Germany; ^8^ German Cancer Research Center (DKFZ) Heidelberg Germany; ^9^ Faculty of Medicine and University Hospital Carl Gustav Carus Technische Universität Dresden Germany; ^10^ Helmholtz‐Zentrum Dresden—Rossendorf (HZDR) Germany

**Keywords:** genotype, high‐grade serous ovarian cancer, phenotype, single cell sequencing, single circulating tumor cells

## Abstract

Single circulating tumor cell (sCTC) analysis enables the determination of predominant CTC phenotypes and genotypes. We previously demonstrated the feasibility of sCTC detection and genomic characterization in high‐grade serous ovarian cancer (HGSOC) by combining immune‐magnetic enrichment and image‐based sorting, followed by whole‐genome amplification (WGA) and next‐generation sequencing‐based copy number alteration analysis (CNA). Here we aimed to improve our workflow by incorporating HGSOC‐specific markers, folate receptor alpha (FRα), and markers to identify epithelial (cytokeratin) and mesenchymal (vimentin) phenotypes for the phenotypic as well as genotypic analysis of sCTCs over the course of treatment in 42 HGSOC patients. We detected a significant reduction of FRα‐positive cells (*P* = 0.0205) and an expansion of cells with a high nuclear staining and no target antigen expression (*P* = 0.002). Before treatment, sCTCs showed an enrichment in CNAs of Chromosomes 2, 7, and 12, while CNA dynamics of sCTCs suggested a potential selection of distinct CNAs specific to the homologous recombination pathway. sCTCs revealed persistent CNAs in the *CDK4* and emerging ones in the *ALK* oncogene. Notably, primary tumors revealed considerable fractions of shared genomic aberrations.

## Graphical abstract

Single circulating tumor cells (sCTCs) from high‐grade serous ovarian cancer patients were enriched, imaged, and genomically profiled using WGA and NGS at different time points during treatment. sCTCs revealed enrichment of alterations in Chromosomes 2, 7, and 12 as well as persistent or emerging oncogenic CNAs, supporting sCTC identity. Notably, primary tumors revealed considerable fractions of shared genomic aberrations.

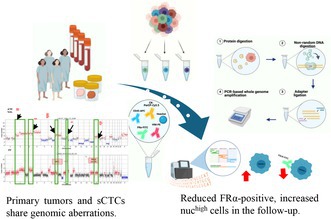

**Abbreviations**
a.Bev: after Bevacizumab treatment
CK: cytokeratin
CNA: copy number alteration
CTX: after platinum‐based chemotherapy.
FRα: folate receptor alpha
HGSOC: high‐grade serous ovarian cancer
NGS: next‐generation sequencing
PT: primary tumor
sCTCs: single circulating tumor cells
WGA: whole‐genome amplification

## 1 Introduction

With 207 252 OC‐related deaths, ovarian cancer (OC) is the deadliest gynecologic malignancy worldwide, accounting for 2.1% of all cancer deaths [1]. As early symptoms are missing, OC is diagnosed at later stages, with high‐grade serous OC (HGSOC) as the most frequently detected subtype and complete tumor resection remaining the most significant independent prognostic factor for overall survival (OS) [2]. Subsequently, applied therapy includes platinum‐based chemotherapy (CTX) [3] and the anti‐angiogenic antibody Bevacizumab [4, 5, 6, 7]. Poly‐ADP‐Ribose‐Polymerase inhibitors (PARPi) in BRCA‐mutated and homologous repair deficient (HRD) tumors were recently shown to improve outcomes significantly [8, 9, 10, 11]. Despite these approaches, the majority of patients will ultimately relapse due to the development of platinum resistance [3, 12] resulting in a relative 5‐year survival rate around 30–40%, which drops down to approximately 20% in higher tumor stages [13]. Consequently, there is an urgent need for more information on disease progression and its driving forces.

It is well known that OC is characterized through a global genomic instability, showing a broad range of known copy number alterations (CNAs), CCNE1‐, MYC‐, and MECOM‐amplifications, PTEN‐, RB‐, and NF1‐deletions, and TP53 mutations (96%) as well as recurrent BRCA, NF1, RB1, and CDK12 mutations [14, 15, 16]. Since tumor tissue is only available at first diagnosis, blood is frequently used across multiple cancer types as a minimally invasive disease monitoring tool in order to understand disease progression.

In this regard, circulating tumor cells (CTCs) have been the most frequently studied blood components in OC with their prognostic relevance clearly shown over the last decade [17, 18, 19, 20, 21] and recently enrolled in a meta‐analysis [22]. Comparing 19 studies from different data bases, the presence of CTCs was significantly associated with an increased risk for disease progression, shorter progression free survival (PFS), OS as well as platinum resistance. Phenotyping and/or sequencing of CTCs in OC revealed morphological variations in marker expression; consequently, relying on the expression of a single marker for CTC identification may underestimate CTC heterogeneity, ubiquity as well as diversity [23]. In this context, a very recently published pilot study, including OC patients, demonstrated that CTC morphology can vary significantly between cancer cells' phenotypes and even their tissue of origin. Putatively, it can also depend on the separation method, timepoint during the treatment, general interpatient variance or pathological parameters, such as tumor grading [24]. Finally, the prevalence of CTC‐clusters in a small subset of OC patients was recently demonstrated using gravity‐based microfiltration [25] and an earlier OC study comprising 2–30 cells in 59% of EOC patients linked their presence to platinum resistance [26]. In this context, we demonstrated that the negative prognostic impact of CTCs was associated with CTCs expressing the resistance related endonuclease ERCC1, which was an independent predictor for PFS and OS [18, 19] and confirmed by Obermayr et al., who identified cyclophilin C gene (PPIC) overexpressing CTCs associated with platinum resistance [27]. Of note, platinum‐based chemotherapy seems to select for CTCs in epithelial–mesenchymal transition (EMT), probably reflecting clonal tumor evolution toward therapy resistance [17, 18, 20, 28, 29].

Most of the CTC studies in OC were performed in CTC‐enriched fractions, providing only limited information of their heterogeneous features and their impact on disease progression, often due to low detection rates, dependent on the method used [30]. To improve CTC detection rates, researchers now, on the one hand, aim to establish OC‐specific CTC identification markers, including folic acid as well as Pax2 and PAx8 [31, 32, 33]. On the other hand, in order to develop reliable detection tools to understand metastasis and evolutionary drivers of individual CTC genomes, many groups are now moving into single (s) CTC analysis to determine predominant CTC phenotypes as well as genotypes. While sCTC analysis has been successfully performed in other cancer entities using the DEPArrayTM system [34], fluorescence‐activated cell sorting (FACS) [35] or micromanipulation [36, 37], only a very few groups addressed this topic in OC [38, 39].

In a small pilot study, we recently demonstrated the feasibility of sCTC detection and genomic characterization in HGSOC using negative immunomagnetic enrichment, followed by immunofluorescence staining and imaging for Hoechst, ERCC1, CD45, CD11b, and cytokeratin (CK) sCTC sorting with the DEPArrayTM NxT [40]. Subsequent whole‐genome amplification (WGA) and next‐generation sequencing (NGS)‐based CNA analysis resulted in the detection of three different sCTC subtypes, interpatient heterogeneity of phenotypically similar sCTCs, as well as intrapatient heterogeneity of phenotypically different sCTCs; however, an aberrant character was only confirmed in about 10% of sCTCs.

We here markedly improved our experimental workflow by incorporating HGSOC‐specific markers, namely the folate receptor alpha (FRα) and markers to identify epithelial (CK) and mesenchymal [vimentin (Vim)] phenotypes for the phenotypic as well as genotypic analysis of sCTCs over the course of treatment in 42 HGSOC patients. In addition, we compared sCTC characteristics with those of the matched primary tumors (PTs) to reveal differences/similarities between their copy number (CN) profiles.

It was the purpose of our study to give insights into the clonal evolution of OC during therapy to use this knowledge for the identification of biomarkers to predict prognosis or response to the given therapy as well as personalized therapy approaches.

## 2 Methods

### 2.1 Characterization of study patients

**Fig. 1:**
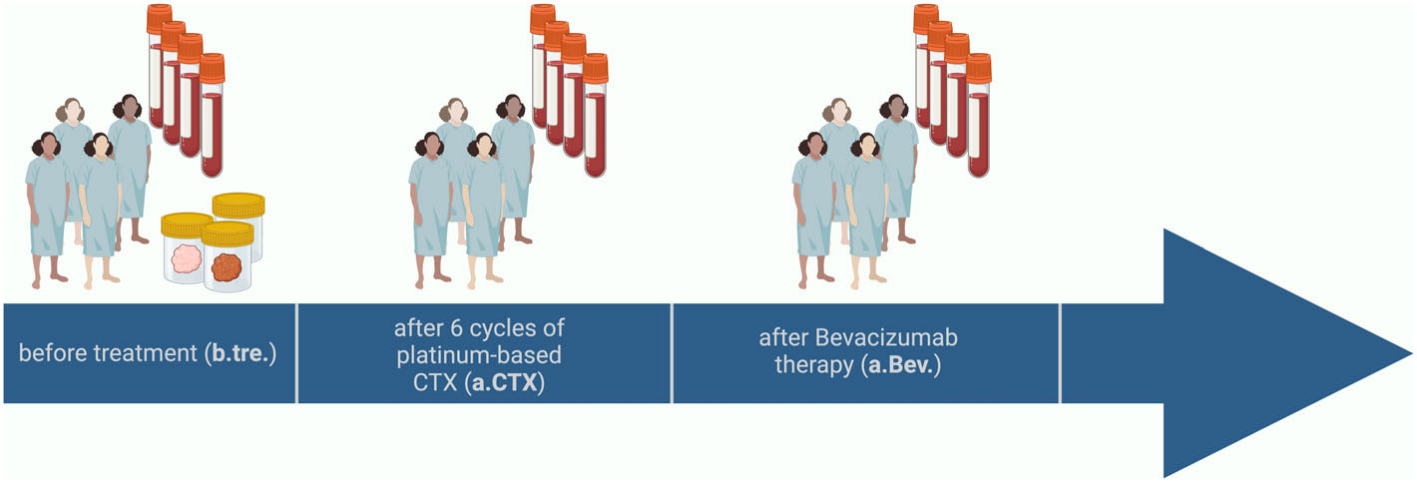
Single circulating tumor cell screening time points of high‐grade serous ovarian cancer patients' blood. Created with biorender.com.

The present study was conducted at the Department of Gynecology and Obstetrics at the University Hospitals of Essen and Dresden, Germany, with the latter group only providing patient blood and tumor samples. In total, 55 patients presenting with first diagnosis of HGSOC between January 2020 and February 2023 participated in this study. Tumors were classified according to the WHO classification of tumors of the female genital tract. Grading was conducted using the grading system proposed by Silverberg, and tumor staging was classified according to the Fédération Internationale de Gynécology et d'Obstétrique (FIGO). The whole study population underwent primary radical surgery. Total abdominal hysterectomy, bilateral salpingo‐oophorectomy, infragastric omentectomy, and peritoneal stripping were performed. The most important aim of surgery was to achieve macroscopic complete tumor resection. Pelvic and periaortic lymphadenectomy was only performed in patients with macroscopically affected lymph nodes. Except for some patients who experienced a sudden death, all patients received at least six cycles of carboplatinum AUC 5 and paclitaxel 175 mg·m^−2^. Bevacizumab (15 mg·kg^−1^) was administered every 3 weeks simultaneous to chemotherapy and as maintenance therapy for a total of 15 months for FIGO stages IIIB and higher. PARP inhibitors were applied in 15 patients for the maintenance treatment of HRD‐positive patients after first‐line platinum therapy in combination with Bevacizumab. Detailed information of clinical characteristics as well as treatment for every single patient is given in Table S1. Blood samples taken before treatment (b.tre.) and in the follow‐up of the disease [23/42 after platinum‐based chemotherapy (a.CTX) and 11/23 after subsequent Bevacizumab treatment (a.Bev.)] were investigated for this study (Fig. 1). The mean time difference between the first (b.tre) and second (a.CTX) blood sampling was 5.35 (range 4–8) months, and 15 (range 14–18) months between the first (b.tre.) and third (a.Bev.) sampling. Informed and written consent was obtained from all patients; the study was approved by the Local Ethic Committees (Essen: 17‐7859‐BO; Dresden: EK236082012) and performed according to the declaration of Helsinki.

### 2.2 Cell culture

Two OC cell lines, OVCAR‐3 and SKOV‐3, were used for experiments establishing the FRα protocol. OVCAR‐3 cells were obtained from the American Type Culture Collection (ATCC®, Manassas, VS, USA). SKOV‐3 cells were provided by the Department of Gynecology and Obstetrics of the Carl Gustav Carus University of Dresden, Germany. Both cell lines were thawed from long‐term nitrogen storage and cultured for 2 weeks before use. OVCAR‐3 cells were cultured in RPMI 1640 medium (Gibco; Life Technologies Limited, Paisley, UK) containing 10% fetal bovine serum (Sigma‐Aldrich Chemie GmbH, Steinheim, Germany), 1% penicillin–streptomycin (10,000 U·mL^−1^, Sigma‐Aldrich Chemie GmbH), and 1% L‐glutamine (200 mm, Gibco, Life Technologies Limited). SKOV‐3 cells were cultured in McCoy's 5a (Gibco, Life Technologies Limited) containing 10% fetal bovine serum and 1% penicillin–streptomycin. All cells were cultured at 37 °C with 5% CO2 and sub‐cultured twice weekly. All cell lines have been authenticated in the past 3 years, and all experiments were performed with mycoplasma‐free cells.

### 2.3 Enrichment of sCTCs

**Fig. 2:**
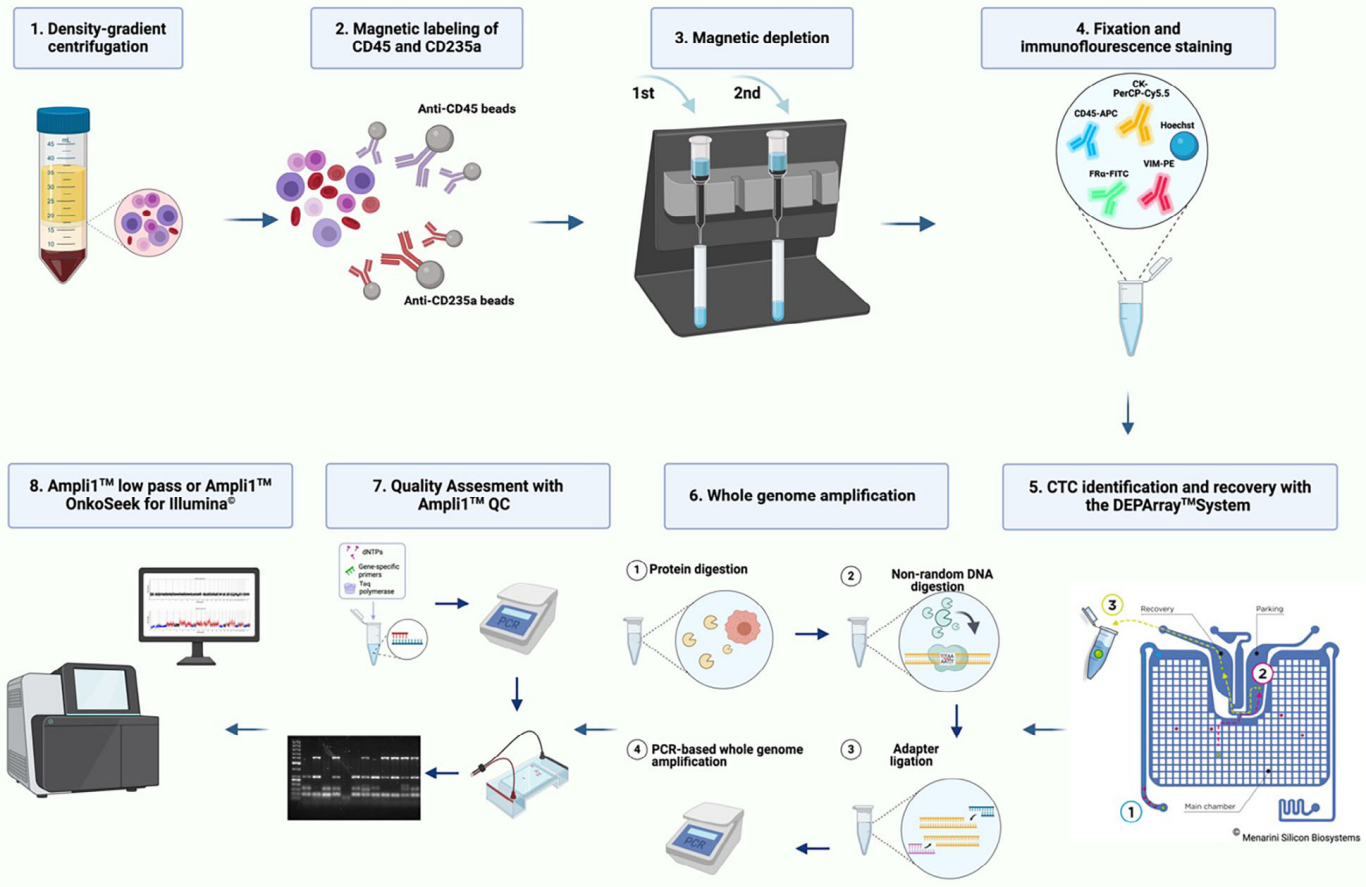
Workflow for single circulating tumor cell isolation and molecular characterization of blood samples from high‐grade serous ovarian cancer patients. Created with BioRender.com.

The general workflow (Fig. 2) has already been described in detail in our previously published study [40] with the exception of the used immunofluorescence staining and the associated DEPArray™ NxT settings which have now been markedly changed and improved. The workflow included a density gradient centrifugation using 10 mL EDTA‐blood, followed by two rounds of depletion of erythrocytes and leukocytes using MACS® columns (Miltenyi Biotec, Bergisch Gladbach, Germany) and MACS® MicroBeads (Miltenyi Biotec) to obtain a CTC‐enriched cell suspension.

### 2.4 Immunofluorescence staining and DEPArray™ NxT settings for sCTC detection

The DEPArray™ NXT system [Menarini Silicon Biosystems (MSB), Bologna, Italy] was applied for fluorescence imaging and sorting. Two phenotypically different OC cell lines, the epithelial‐like OVCAR‐3 cells and mesenchymal‐like SKOV‐3 cells were used to establish FRα‐, Vim‐ and CK‐staining. Each antibody was filtered using Ultrafree®‐MC sterile centrifugation units (0.65 μm; Merck Millipore Ltd., Tullagreen, Ireland) before use. The tandem signal enhancer (human, no.: 130‐099‐888; Milenyi Biotec, Bergisch Gladbach, Germany) was used to prevent nonspecific binding of the tandem fluorophore, particularly to proteins of monocytes. The specialized blocking agent was applied at a dilution of 1 : 9 with autoMACS® RunningBuffer (Milenyi Biotec). Anti‐Vim antibody coupled to PE [Vimentin (D21H3) XP® Rabbit mAb‐ PE conjugate, no.: 12020, Cell Signaling Technology, Bologna, MA, USA] at a 1 : 30 dilution was incubated at 2–8 °C for 10 min, and then, APC‐coupled anti‐CD45 antibody (clone 5B1, 130‐113‐114; Miltenyi Biotec) was added at a 1 : 10 dilution and incubated for another 10 min at 2–8 °C. After washing with 1 mL autoMACS® RunningBuffer, the supernatant was discarded and the cells were permeabilized with 0.1% TitonX (Roth, Karlsruhe, Germany) diluted in autoMACS® RunningBuffer at RT for 20 min. Subsequently, the cells were washed with 500 μL autoMACS® RunningBuffer and centrifuged at 400 ***g*** for 10 min, followed by another washing with 1 mL autoMACS® RunningBuffer. For intracellular staining of CK and FRα, the tandem signal enhancer was added again in a 1 : 10 dilution with 0.1% TitonX in autoMACS® RunningBuffer. Simultaneously, anti‐CK antibody coupled to PerCP‐Cy5.5 at a dilution of 1 : 50 [anti‐pan cytokeratin antibody (PerCP‐Cy5.5), no.: AC12‐0249‐19, Abcore, Ramona, California] and anti‐FRα staining coupled to FITC [anti‐human FOLR1 (FITC), no.: 035711‐FITC, US Biological Life Science, Salem, MA, USA] at a dilution of 1 : 14.3 were incubated at 2–8 °C for 20 min. After washing with 1 mL of 0.1% TitonX in autoMACS® RunningBuffer, another washing step was performed with only 1 mL of autoMACS® RunningBuffer. After having discarded the supernatant, nuclear staining was performed with Hoechst 33342 at RT for 5 min. After the final centrifugation for 10 min at 400 ***g***, the supernatant was discarded and 100 μL autoMACS® RunningBuffer was added. Cells were counted and stored at 2–8 °C for a maximum of 4 days prior to DEPArray™ NxT (MSB) sorting, which was performed according to the manufacturer's protocol. The image settings and detection cutoffs (Tables 1 and 2) for sCTC sorting were set uniformly to allow for comparative phenotypic analysis between patient samples.

**Table 1:** DepArrayTM system chip scan settings used for sCTC detection.

Scan settings	Exposure (ms)	Camera gain	Lamp intensity (%)	Offset (μm)
APC	300	1×	100%	38
PE	300	1×	100%	38
DAPI	25	1×	25%	36
Brightfield	3	1×	5%	26
FITCsb	450	1×	100%	36
PerCp‐Cy5.5	350	2×	100%	38

**Table 2:** Signal thresholds for fluorophore coupled antibodies used in the FRα protocol.

Fluorophore	Positive signal threshold
Hoechst (DAPI‐channel)	≧ 300
CD45^−^ APC	≧ 120
FRα – FITC	≧ 130
Vim‐PE	≧ 160
CK‐PerCp‐Cy5.5	≧ 200

### 2.5 Whole‐genome amplification in sCTCs

WGA was performed according to a single‐tube protocol using adapter–linker PCR based on MseI digestion of the genome [41, 42], which is now commercialized as the Ampli1™ Whole Genome Amplification Kit by MSB. Subsequently, quality assessment was performed using the Ampli1™ QC Kit (MSB). Depending on the estimated Genomic Integrity Index (GII), Ampli1™ LowPass and/or Ampli1™ OnkoSeek (MSB) analysis were performed.

### 2.6 Next‐generation sequencing and data analysis of sCTCs

All potential sCTC with a GII of 2–4 were sequenced using the Ampli1™ LowPass analysis (MSB). CN‐profile analysis of the Ampli1™ LowPass data was performed using the Control‐FREEC software, and ploidy levels were automatically estimated by the MSB pipeline based on the underlying CN levels [43]. Genome‐wide CNAs were used to proof the malignant identity of the isolated sCTCs. A pool of leukocytes from each sample served as control. Deep sequencing was performed with sCTCs with a GII of 3–4 using the Ampli1™ OncoSeek panel (MSB), which covers variants in 60 cancer‐related genes and additionally provides single‐gene CNA data in 19 genes.

Library preparation for both sequencing approaches was performed at MSB, followed by sequencing on an Illumina NovaSeq™ platform. The Integrative Genomics Viewer (IGV) was used to visualize the sequencing data [44], while GraphPad Prism 9.3.1 was used for statistical analysis. Recognizing that CTCs are rare events and that an appropriate mathematical description of the distribution of CTCs is strongly influenced by the method of detection and selection, all statistical tests were nonparametric. Tests of significance for paired samples were performed using the Wilcoxon test, and for unpaired samples, the Mann–Whitney test was applied. The Friedman test was chosen to compare paired samples at all three time points.

### 2.7 Primary tumor tissue preparation

Formalin‐fixed, paraffin‐embedded (FFPE) PT tissue sections (*n* = 21) were used to assess PT DNA in our HGSOC patient cohort. For internal method validation, we decided to apply two different processing approaches for the PT samples, as initial analyses using the DEPArray™ NxT revealed that the low number of tumor cells substantially limited downstream analyses. PT tissue was either rehydrated, stained, and further enriched for tumor cells (CK‐positive cells) using the DEPArray™ NxT or identified by the pathologist who subsequently extracted DNA using the QIAamp® DNA Micro Kit (Qiagen, Hilden, Germany).

For the first approach prior to sorting with the DEPArray™ NxT, the FFPE material was rehydrated and dissociated using the FFPE Tissue Dissociation Kit (no.: 130‐118‐052, Milenyi Biotec) according to the manufacturer's protocol. After the final step of the protocol, the supernatant was discarded and 1 mL ice‐cold PBATw buffer [comprising 0.5 g Tween‐20 (no.: P9416; Sigma‐Aldrich, St. Luis, MO, USA), 10 g BSA, (Sigma‐Aldrich Chemie GmbH, Steinheim) in 1000 g PBS (Gibco, Life Technologies Limited)] was added. A total of 500 000 cells were added to 1 mL PBATw buffer and centrifuged at 1000 ***g*** at 4 °C for 5 min. After having discarded the supernatant, the cells were incubated in 500 μL PBATw buffer for 10 min at RT. After a further centrifugation for 5 min at 1000 ***g*** and 4 °C, the supernatant was removed and 80 μL PBATw buffer was added to the sample. FITC‐labeled anti‐CK antibody [Anti‐Cytokeratin‐FITC (clone REA831), no.: 130‐112‐743, Milenyi Biotec] and APC‐labeled anti‐Vim antibody [Anti‐Vimentin‐APC (clone REA409), no.: 130‐118‐361, Milenyi Biotec] were added at a 1 : 10 dilution and incubated at 2–8 °C for 20 min. Subsequently, 1 mL PBATw buffer was added and the sample was centrifuged at 1000 ***g*** at 4 °C for 5 min. After removal of the supernatant, the washing step was repeated and 495 μL PBATw buffer and 5 μL DNA staining solution [100× DNA staining solution, conc. 1000 μm, DAPI (Sigma‐Aldrich, St. Luis) in nuclease‐free water (no.: 10977035, Invitrogen, Carlsbad, California)] were incubated for 30 min at RT. After the addition of 1 mL autoMACS® RunningBuffer and centrifugation at 1000 ***g*** for 5 min, the supernatant was removed and the washing step was repeated. Subsequently, 100 μL autoMACS® RunningBuffer were added and the cells were counted. Prior to DEPArray™ NxT sorting, samples were prepared according to the manufacturer's protocol and sorting was performed using the FFPE‐RUO application and image settings were adjusted as necessary. CK‐positive tumor cells were sorted as a pool, in addition to Vim‐positive cells (stromal cells) which served as a control.

### 2.8 Next‐generation sequencing and data analysis of FFPE tissue

To generate sufficient templates for sequencing, a WGA was performed with each DEPArray™ NxT sample due to the low cell count. The DEPArray™ LysePrep Kit (no.: KI0048; MSB) was used to ensure complete digestion of the cellular material; all other WGA steps were performed as previously published [40] and described above. Due to the different DNA preparations, two sequencing pipelines were used. The WGA amplified DEPArray™ NxT samples were prepared with the Ampli1™ LowPass Kit for Illumina® (no.: KI0123, no.: KI125; MSB) to be subsequently processed with the Ampli1™ LowPass pipeline. The extracted DNA from the pathologists was prepared for sequencing using the DEPArray™ LibPrep Kit for Illumina® (no.: KI0015, no.: KI0014; MSB). Both library preparations and subsequent sequencing on an Illumina NovaSeq™ platform were performed by MSB.

## 3 Results

### 3.1 Phenotypic evaluation of sCTCs

**Fig. 3:**
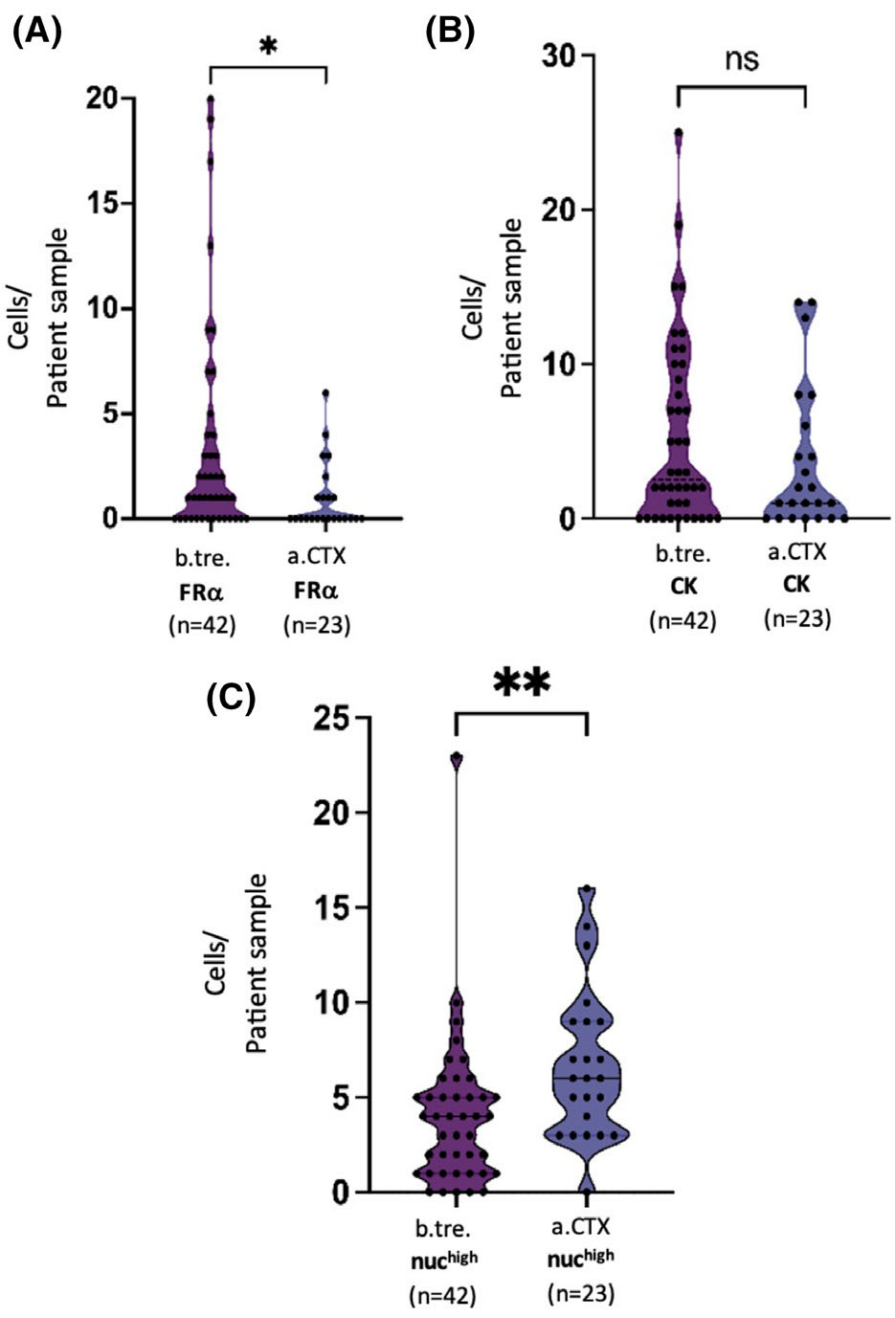
(A) Violin plot of FRα‐positive cells in unmatched HGSOC patients b.tre. (*n* = 42) and a.CTX (*n* = 23). (B) Violin plot of CK‐positive cells in unmatched HGSOC patients b.tre. (*n* = 42) and a.CTX (*n* = 23). (C) Violin plot of nuc^high^ cells in unmatched HGSOC patients b.tre. (*n* = 42) and a.CTX (*n* = 23). Bars indicate median and quartiles. Statistical analysis between the two unmatched time points was performed using the Mann–Whitney test and between the three matched time points using the Friedman test (ns = nonsignificant, *P ≤ 0.05, **P ≤ 0.01, ***P ≤ 0.001, ****P ≤ 0.0001). before treatment (b.tre.); after CTX (a.CTX); after Bevacizumab (a.Bev.); cytokeratin (CK); folate receptor alpha (FRα); high‐grade serous ovarian cancer (HGSOC).

**Fig. 4:**
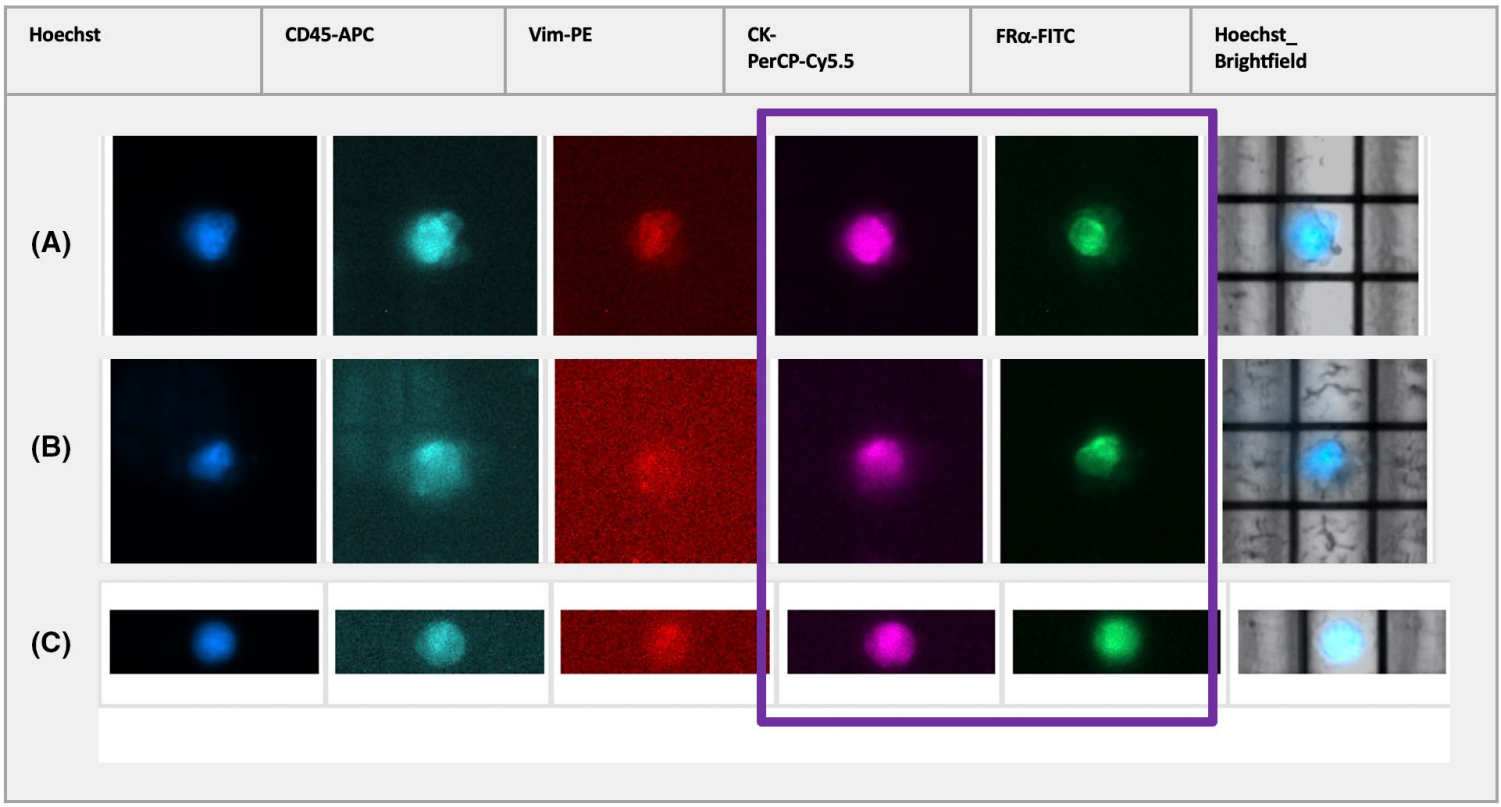
Fluorescence imaging of double‐positive CK‐positive and FRα‐positive cells in Patient 25 (A) and Patient 36 (B) b.tre. and in Patient 27 (C) a.CTX treatment using the DEPArray™ System. Brightfield‐visible DEP cage can be used as a reference point, as scan protocols lacked a scale bar. before treatment (b.tre.); after CTX (a.CTX); cytokeratin (CK); folate receptor alpha (FRα).

**Fig. 5:**
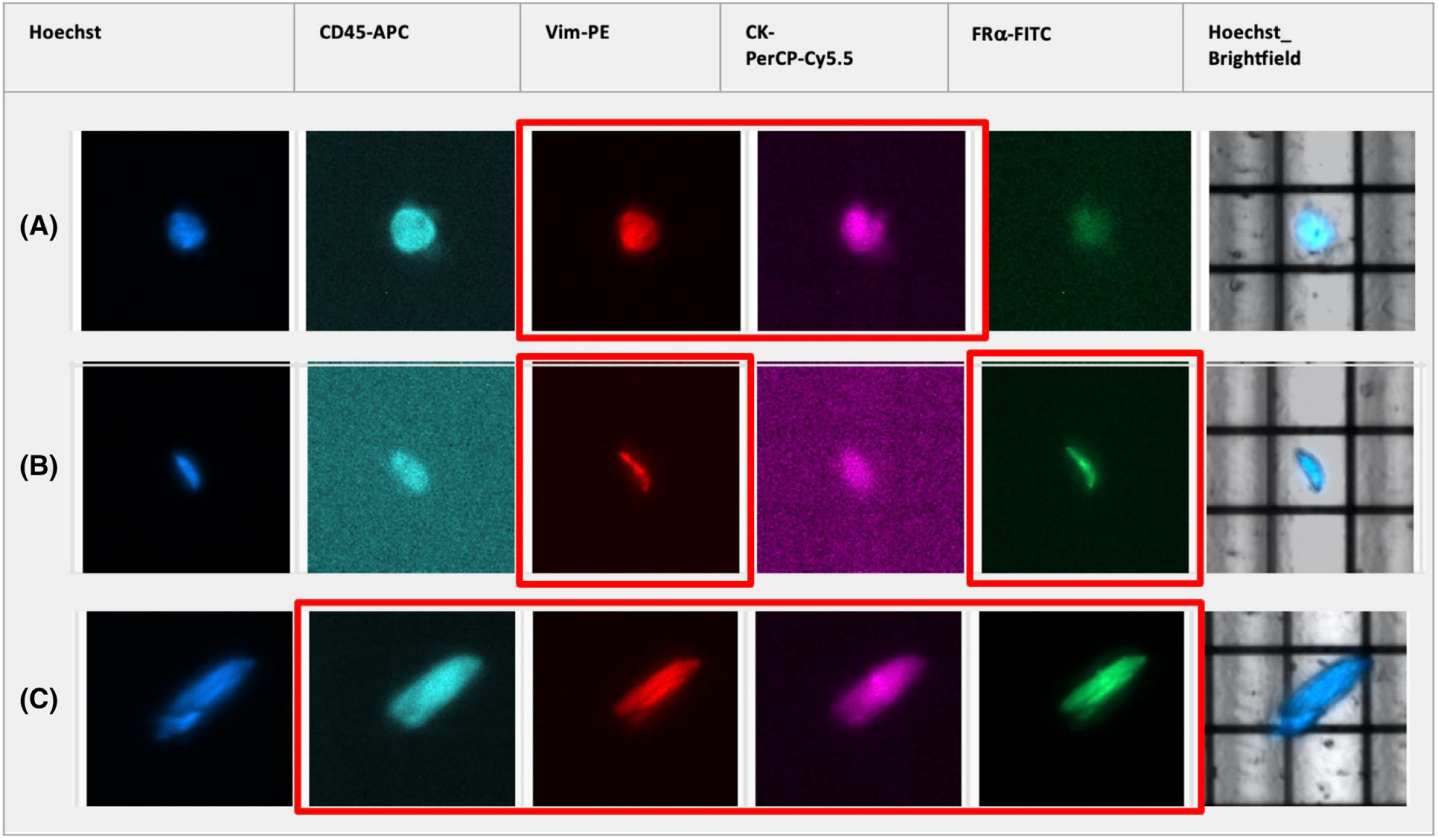
Vim‐positive cells imaged by the DEPArray™ System. (A) A single cell positive for CK and Vim in Patient 41 b.tre. (B) A single cell positive for FRα and Vim in Patient 50 b.tre. and (C) a single cell positive for CD45, CK, Vim, and FRα in Patient 29 b.tre. Brightfield‐visible DEP cage can be used as a reference point, as scan protocols lacked a scale bar. before treatment (b.tre.); after CTX (a.CTX); cytokeratin (CK); folate receptor alpha (FRα); vimentin (Vim).

Different sCTC phenotypes were detected in our patient cohort before therapy and in the course of the disease. Figure 3 illustrates the dynamics for FRα‐, CK‐and nuc^high^‐positive cells. Whereas no significant changes were observed for the detection of CK‐positive cells after the given therapies (Fig. 3B), a significant (*P* = 0.0205) reduction in FRα‐positive cells was found after chemotherapy (Fig. 3A), which was also confirmed in matched samples. Interestingly, an increased number of sorted cells with no detectable antigen expression but high nuclear staining intensity (above 1500), further defined as nuc^high^ cells, were detected before treatment and expanded significantly (*P* = 0.0004) during the course of the disease (Fig. 3C). Of note, these accounted for > 20% among our used OC cell lines compared with 0.66% of healthy donor cells. Images illustrating cell morphology will not be analyzed with respect to morphological features, as the morphological data were obtained exclusively via the imaging capabilities of the DepArray system, which are not suitable for detailed morphological analysis and are not comparable to data generated by advanced imaging modalities, such as confocal fluorescence microscopy. Moreover, a low number of CK‐/FRα‐double‐positive cells was detected; however, no significant changes between the respective time points were noted (Fig. 4). Of note, these cells represented only a minor fraction of the total detected cell population. Due to their low abundance, statistical or meaningful analytical evaluation was not possible. Vim is also expressed in white blood cells and thus, a high frequency of Vim‐positive cells was detected in each patient. Consequently, they were only sampled exemplarily for genetic analysis and no evaluation of the phenotypic dynamics was performed. Noteworthy, CK‐ and Vim‐double‐positive cells (Fig. 5) as well as FRα and Vim‐double‐positive cells (Fig. 5B) were detected. Furthermore, cells with signal combinations of FRα, CK as well as Vim (Fig. 5C) were observed in individual cases but did not represent reoccurring events in our HGSOC patient cohort.

### 3.2 Genotypic evaluation

#### 3.2.1 SC quality investigations

**Fig. 6:**
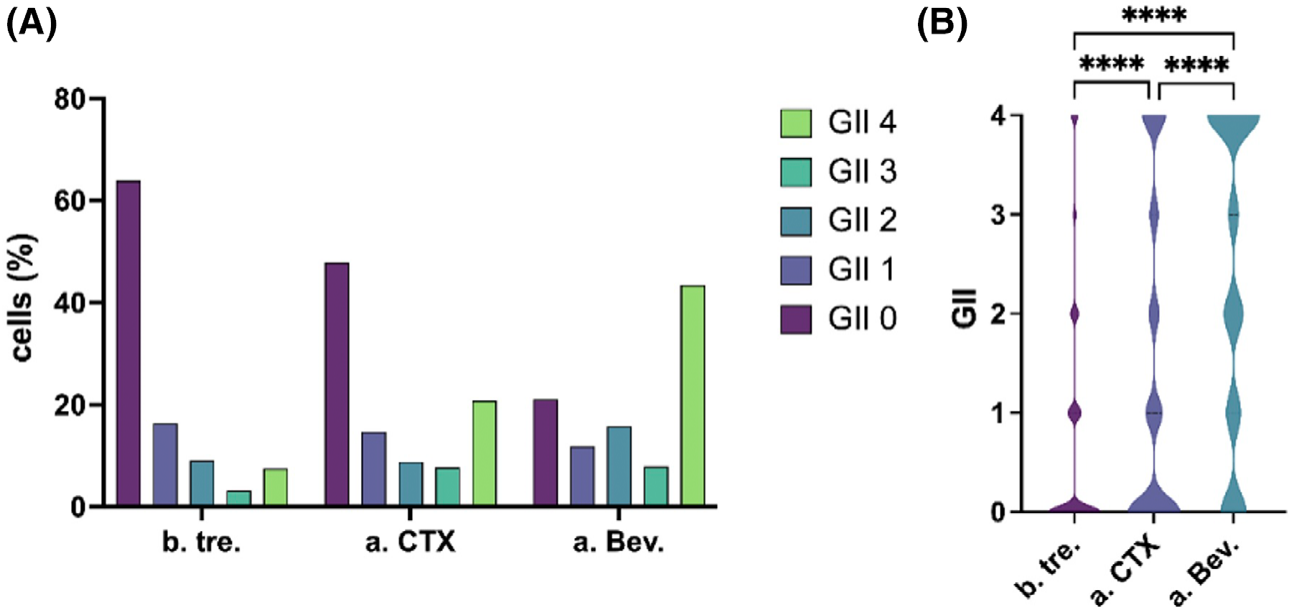
(A) Bar chart visualizing the Genomic integrity index (GII) distribution of all 709 single cells classified by their different sampling time points (*n* = 42 patients b.tre.; *n* = 30 patients a.CTX; *n* = 12 patients a.Bev.) (B) Violin plot with statistical evaluation of the GII distribution of all 709 single cells. Bars indicate median and quartiles. Statistical evaluations were performed with the Kruskal–Wallis test (ns = nonsignificant, *P ≤ 0.05, **P ≤ 0.01***P ≤ 0.001****P ≤ 0.0001) before treatment (b.tre.); after CTX (a.CTX); after Bevacizumab (a.Bev.).

In total, the genomic quality of 709 potential sCTCs (*n* = 246 FRα‐positive cells; 187 CK‐positive cells; *n* = 163 nuc^high^ cells) from 84 samples obtained over the course of treatment were assessed. Since the criteria for identification of the different cell types were uniform across samples, quality assessment of different cell types was possible. In total, 31.31% (222/709) of cells with a GII between 2 and 4 were selected for sequencing, whereas 68.69% (487/709) had no sufficient quality for sequencing. Their quality distribution across the different time points is shown in Fig. 6A. In general, a significant increase in genomic quality that improved with ongoing treatment was noted, which was further confirmed for every CTC‐subtype during the course of the disease (Fig. 6B, Fig. S1A–C).

#### 3.2.2 LowPass CNA data in sCTCs and matched primary tumors of HGSOC patients

**Fig. 7:**
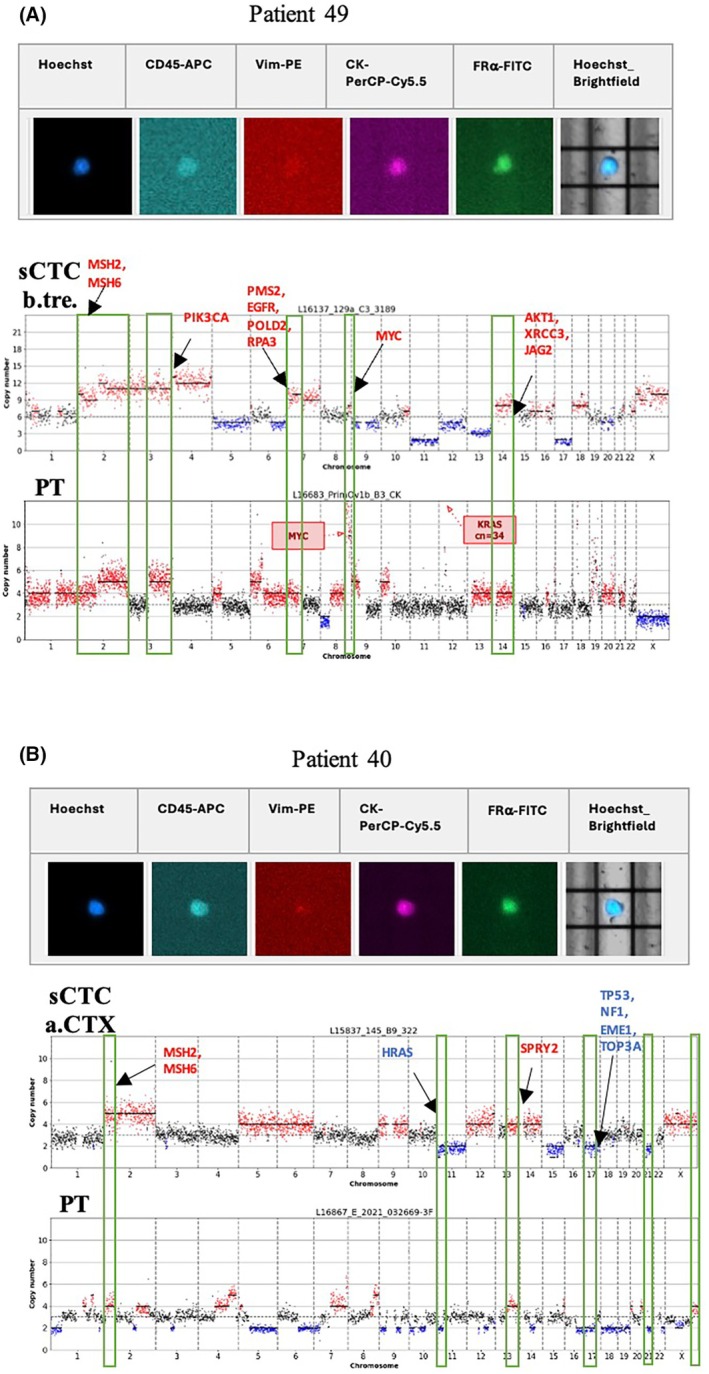
(A) sCTC CN profile b.tre. and matched PT tissue CN profile from Patient 49 (PT CN profile was obtained using DEPArray™ NxT FFPE‐PT tissue sorting approach). (B) sCTC CN profile a.CTX and matched PT tissue CN profile from Patient 40 (PT CN profile was obtained using the pathologist FFPE‐PT identification approach). Green boxes mark similarities between CN profiles. Brightfield‐visible DEP cage can be used as a reference point, as scan protocols lacked a scale bar. before treatment (b.tre.); after CTX (a.CTX); after Bevacizumab (a.Bev.); copy number (CN); formalin‐fixed paraffin‐embedded (FFPE); single circulating tumor cell (sCTC); primary tumor (PT).

Two approaches were chosen to evaluate the CN profile of the PT DNA. In total, 21 HGSOC FFPE‐PT samples were available for investigation, of which five were processed applying both methods. In general, both methods were feasible for the analysis of HGSOC FFPE‐PT. While the DEPArray™ sorted material generated a more differentiated CN profile, the pathologist's identification approach was also suitable for the CNA comparison between the PT and sCTCs (data not shown). A total of 289 potential sCTCs were sequenced for the detection of CNAs, 19/289 (6.57%) originating from 13 patient samples (26%) showed CNAs expected from cells with a tumor origin. The heterogeneous nature of sCTCs as well as their limited number did not allow a correlation of phenotypes and genotypes. Nevertheless, CNA analysis of the PT tissue in nine patients allowed for comparative CNA analysis to demonstrate PT origin. Fig. 7A demonstrates similarities (green boxes) between one sCTC b.tre. and the matched PT of Patient 49, while Fig. 7B displays similarities between a sCTC a.CTX and the matched PT of Patient 40. Although common features were documented between the PT and sCTCs, irrespective of the sampling time point, diversity dominated. However, the detected CNA similarities supported the PT origin of the sCTCs. Interestingly, the sCTCs b.tre. shared several large CNAs with the matched PT, while the sCTCs a.CTX seemed to share fewer features.

#### 3.2.3 Common CNAs in sCTCs of HGSOC patients at different time points

**Fig. 8:**
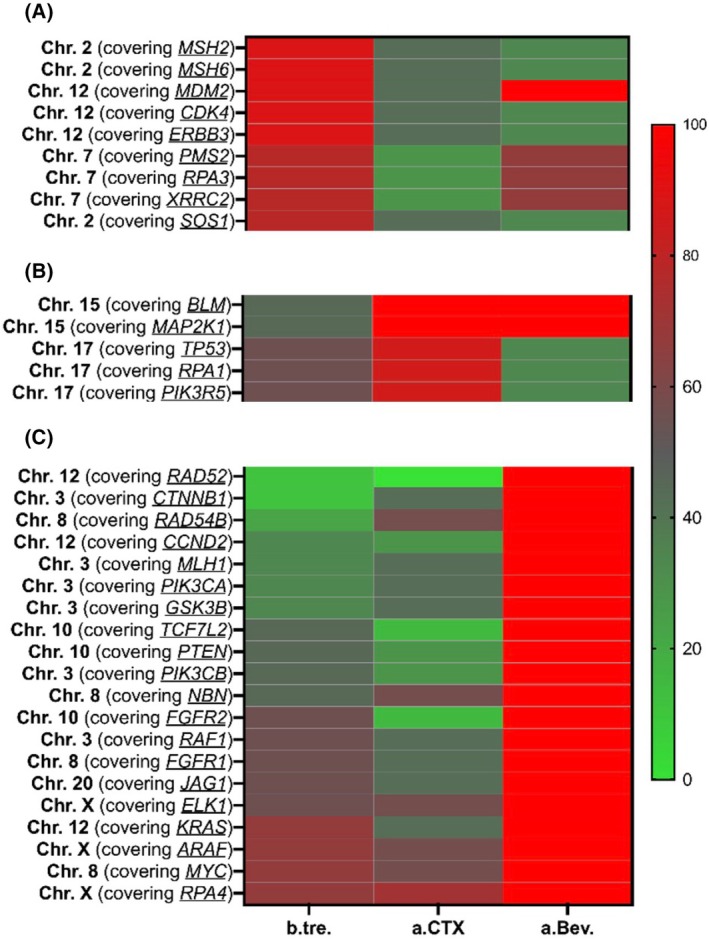
Single circulating tumor cell loci ordered by high CNA detection at the different time points from high‐grade serous ovarian cancer patients. (A) b.tre., (B) a.CTX and (C) a.Bev before treatment (b.tre.); after CTX (a.CTX); after Bevacizumab (a.Bev.); copy number alteration (CNA).

For 19 sCTCs (*n* = 9 b.tre., *n* = 7 a.CTX, *n* = 3 a.Bev. treatment), sequencing was feasible. Due to the apparent heterogeneity of CNAs among sCTCs, the analysis focused on the frequency in which the aberrations occurred within chromosomal segments of a certain size (> 8000 kBp), regardless of CNA type (amplification/deletion). The aim was to elucidate oncogenes present in these CNA segments to gain a deeper understanding of commonly affected cancer signaling pathways. The heatmaps in Fig. 8 visualize the chromosomal loci frequently affected (red) by CNAs in sCTCs b.tre. (Fig. 8A), a.CTX. (Fig. 8B), and a.Bev. (Fig. 8C). Chromosomal loci with enriched CNAs are colored red, while a normal CN is represented by green. Within each heatmap, the status of the same segment at the other time points is also displayed to reveal the CNA dynamics over time. In sCTCs b.tre. (Fig. 8A), CNAs were enriched in Chromosomes 2, 7, and 12. The CNA in Chromosome 12 affected multiple signaling pathways, namely P53 (*MDM2*)‐, RB (*CDK4*)‐, and RTK/RAS (*ERBB3*) signaling. The evaluation of enriched CNAs a.CTX (Fig. 8B) revealed common CNAs between sCTCs in Chromosomes 15 and 17, while the CNAs a.Bev. treatment were distributed across the genome (Fig. 8C), which might be due to the small sample size. Changes in Chromosome 15 are noteworthy, due to an increase that was consistently observed at the two later time points, suggesting favorable characteristics within Chromosome 15. Normal CNs were detected across multiple chromosomes at every time point (Fig. S4).

Although very preliminary and observational, CNA changes between sampling time points were mainly identified in genes involved in the KEGG (RAD52 and RAD54B) for homologous recombination and endometrial cancer (CTNNB1, MAP2K2, and PIK3R2) signaling pathway, rarely at primary diagnosis but increasingly affected in the course of the disease (data not shown). Moreover, CNAs detected in about a half of sCTCs b.tre and a.CTX were mainly detected in genes of the KEGG for homologous recombination (BRCA2 and RAD51C) and KEGG for endometrial cancer pathway (CTNNA2, FOXO3, SOS2, and TCF7L1), probably highlighting a potential selection of specific CNAs in the course of platinum‐based chemotherapy.

#### 3.2.4 Intrapatient sCTC heterogeneity

**Fig. 9:**
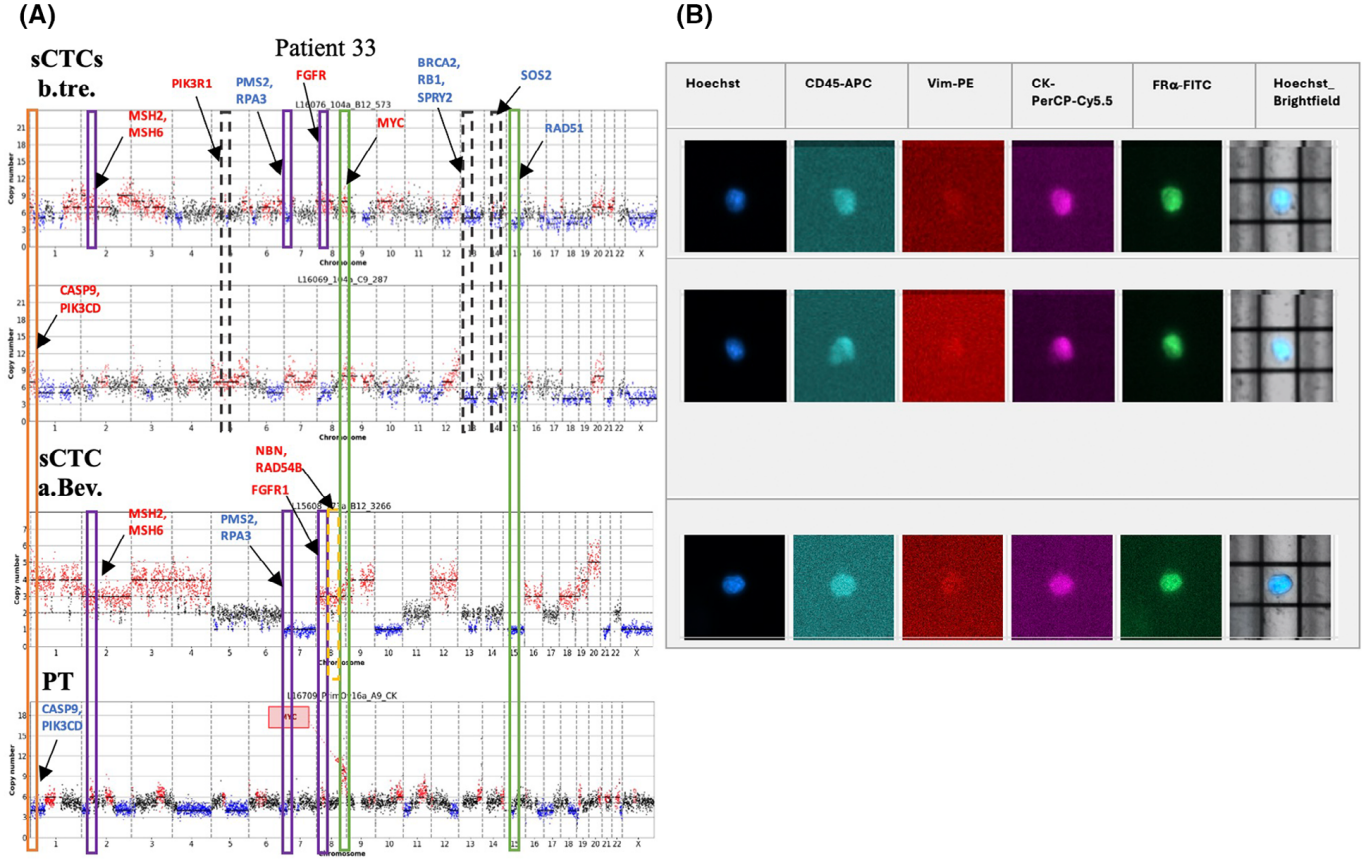
Two sCTCs CN profiles b.tre. and one sCTC CN profile a.Bev. with matched PT tissue CN profile from Patient 33. PT CN profile was obtained using the DEPArray™ NxT FFPE‐PT tissue sorting approach. Green boxes mark similarities between all CN profiles, purple boxes mark similarities between some of the investigated CN profiles, and orange boxes mark differences. Brightfield‐visible DEP cage can be used as a reference point, as scan protocols lacked a scale bar. a.Bev., after Bevacizumab; a.CTX, after CTX; b.tre., before treatment; CN, copy number; FFPE, formalin‐fixed paraffin‐embedded; PT, primary tumor; sCTC, single circulating tumor cell.

We would like to describe two HGSOC patients with sequencing data available from the PT as well as sCTCs at different sampling time points, illustrating sCTC heterogeneity during treatment. Figure 9 displays the CN profiles of all detected sCTCs and the matched PT of Patient 33. Notably, the frequent change between amplifications and deletions was observed in both sCTCs b.tre and the PT. Some amplifications (green boxes) detected in the PT were also detected in all sCTCs. This affected Chromosome 8, covering the *MYC* gene, as well as the deletion in Chromosome 15, covering the *RAD51* gene. Interestingly, the PT presented with a deletion on Chromosome 1, covering *CASP9* and *PIK3CD*, while all detected sCTCs presented with amplifications at this location (orange box). In addition, we could detect CNAs present in the PT but heterogeneously present in the sCTCs b.tre (purple boxes), namely the amplification on Chr.2 (*MSH2* and *MSH6*) and Chr.8 (*FGFR1*) as well as the deletion on Chr.7 (*PMS2* and *RPA3*). When only comparing sCTCs, CNAs newly detected a.Bev treatment (yellow box) affected segments on Chromosome 8 (*NBN* and *RAD54B*) as well as other segments also encompassing genes of the HR pathway (data not shown). CNAs affecting Chr.5 (*PIK3R1*), Chr.13 (*BRCA2, RB1*, and *SPRY2*), and Chr.14 (*SOS2*) were detected in both pretreatment sCTCs (gray boxes) but were no longer detectable in the sCTC a.Bev treatment.

**Fig. 10:**
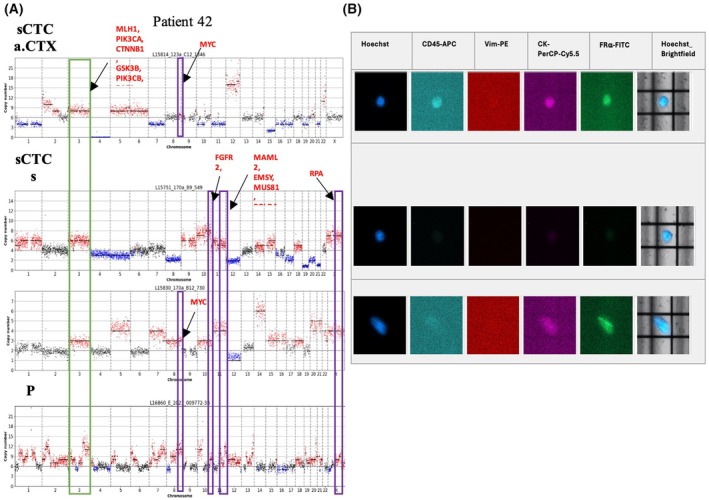
One sCTC CN profile a.CTX and two sCTCs CN profiles a.Bev. with matched PT tissue CN profile from Patient 42. PT CN profile was obtained using the pathologist FFPE‐PT identification approach. Green boxes mark similarities between all CN profiles, purple boxes mark similarities between some of the investigated CN profiles, and orange boxes mark differences. Brightfield‐visible DEP cage can be used as a reference point, as scan protocols lacked a scale bar. a.Bev., after Bevacizumab; a.CTX, after CTX; b.tre., before treatment; CN, copy number; FFPE, formalin‐fixed paraffin‐embedded; PT, primary tumor; sCTC, single circulating tumor cell.

The results obtained for Patient 42 are shown in Fig. 10, revealing shared amplifications between the PT and all sCTCs (green box) on Chromosome 3, covering *MLH1*, *PIK3CA*, *CTNNB1*, *GSK3B*, *PIK3CB*, and *RAF1*, respectively. Besides the detection in the PT, the amplification covering the *MYC* gene on Chromosome 8 was also identified in the sCTC b.tre. and in one sCTC a.Bev treatment (purple box). Amplifications on Chromosome 10 (*FGFR2*, *TCF7L2*), Chromosome 11 (*MAML2*, *EMSY*, *MUS81*, *POLD4*, and *BAD*), and Chromosome X (*RPA4*) were identified in the PT and both sCTCs a.Bev treatment but not in the sCTC b.tre. (purple boxes). Notably, those amplifications covered multiple genes involved in RTK/RAS signaling (*FGFR2*, *MUS81*, *POLD4*, and *RPA4*).

#### 3.2.5 Deep sequencing data in sCTCs

We were able to use 14 sCTCs for single‐gene CNAs as well as variants by the Ampli1™ OnkoSeek analysis and detected CNAs affecting some of the investigated genes in 7/14 sCTCs (*n* = 5 b.tre. from three patients; *n* = 2 a.CTX from two patients).

While some Ampli1™ LowPass CNAs were confirmed by the Ampli1™ OnkoSeek analysis (data not shown), discrepancies in results due to the different methods were noted. Single‐gene CNAs detected by the Ampli1™ OnkoSeek analysis revealed a significant increase in the CN of *CDK4* (Chr.12), *FGFR3* (Chr. 4), *EGFR* (Chr.7), and *FGFR2* (Chr.10) in sCTCs b.tre. A significant increase in the CN of *ALK* (Chr.2), *PIK3CA* (Chr. 3), *CDK4* (Chr.12), and *KRAS* (Chr.12) was detected a.CTX. Moreover, very few copies of the oncogenes *FGFR3* (Chr.4), *KIT* (Chr.4), and *PDGFRA* (Chr.4) were detected in sCTCs a.CTX. Notably, a CN deletion b.tre. and an amplification a.CTX was only detected for the *ALK* oncogene, while an amplification b.tre. and a subsequent deletion a.CTX was only detected for the *FGFR3* oncogene. A constant amplification in pre‐ and post‐CTX sCTCs from different patients was only detected in the *CDK4* oncogene (Fig. S2).

Single‐nucleotide variants were detected in nine sCTCs (*n* = 6 b.tre. from five patients; *n* = 3 a.CTX from two patients) (Fig. S3). *TP53* was the gene most frequently affected by single‐nucleotide variants (SNVs) (COSM11333, COSM43968, COSM44986, and COSM44996). Moreover, SNVs in the *RB1*, *PTEN* (COSM5109), *MYC*, and *ATM* (COSM21825) genes were detected. However, the SNV detection rate in sCTCs and the detection of variant replicates in different sCTCs was rare.

## 4 Discussion

In the current study, we were able to expand our knowledge on sCTC characteristics in the course of treatment in HGSOC. While our previously published study only presented a snapshot of sCTC inter‐ and intrapatient heterogeneity right at primary diagnosis of the disease [40] we here markedly improved our experimental workflow for the phenotypic and genotypic characterization of sCTCs over the course of treatment in 42 HGSOC patients. We further performed comparison studies with the PT to reveal differences as well as similarities between their CN profiles.

Our main findings identified FRα as one marker to select sCTCs appropriately. While other phenotypes did not significantly change over time or were only present anecdotally, nuc^high^ sCTCs were frequently detected; they expanded over time and harbored a good genomic quality for further sequencing. Comparison studies between sCTCs and their matched PT tissue highlighted the tumor origin by revealing common CNA features. Although only a subset of sCTCs fulfilled the genetic requirements to be further processed, *TP53* was the gene most frequently affected by variants in these cells. It seemed as if sCTCs at primary diagnosis were enriched in CNAs of Chromosomes 2, 7, and 12, while post‐treatment sCTCs highlighted the potential selection of favorable CNAs within the HR pathway. Preliminary results led to the speculation that CNAs in the *CDK4* oncogene persist and CNAs in the *ALK* oncogene emerge over time.

### 4.1 sCTC phenotype dynamics in HGSOC patients

In OC, various efforts were taken to select and characterize CTCs for their prognostic, predictive, and monitoring value. Except for a very few studies with small patient numbers, CTC studies in OC were performed using the bulk of CTCs, resulting in different detection rates of CTCs even though similar enrichment and detection methods were applied [22, 23, 30, 38, 39]. To identify the heterogeneous CTC population in HGSOC, studies are now focusing on methods which combine the selection of epithelial and mesenchymal CTCs [45, 46].

Since only a very few data were published for sCTC analysis in OC, our results can only be compared with results obtained in the bulk of CTCs in OC or with sCTC studies in other tumor entities. Although limited success in identifying CTCs based on EpCAM and/or CK was noted for OC [21, 27, 28, 47], we still included CK in our workflow as one major component of epithelial cells. However, besides the detection of CK‐positive sCTCs before, during, and after treatment, our low overall detection rate reinforces the notion that CK alone is not an appropriate marker for sCTC identification in HGSOC, as already shown for the bulk of CTCs [20, 28, 48]. Furthermore, our genetic analysis demonstrated that most of the CK‐positive cells were of poor genomic quality to be further processed. Thus, without elucidating their genetic nature, the value of these cells with regard to their role as a reliable biomarker remains questionable.

In contrast to EpCAM and CK, the FRα has emerged as a new promising biomarker and target in OC. Based on its unique features, new drugs were designed for innovative treatment options, including monoclonal antibodies against FRα, folate–drug conjugates, and antibody–drug conjugates (ADCs). In this context, one FRα‐targeting ADC was recently approved by the US Food and Drug Administration (FDA) for treating patients with FRα‐positive platinum‐resistant OC, fallopian tube cancer (FTC), or primary peritoneal cancer who had previously received one to three systemic anticancer treatments [49]. We here confirm that FRα seems to be a good biomarker for sCTC selection and characterization to monitor HGSOC. Our suggestions are in line with results of *in vitro* experiments from Siwowska et al., who showed cisplatin sensitivity of FRα‐expressing SKOV‐3 cells [50]. Moreover, an unchanged FRα expression was shown in PT samples at interval debulking surgery during CTX treatment [51], assuming that tumor cells do not lose FRα expression during current treatment regimens, which also supports that our reduction in FRα‐positive cells could be a true reduction in sCTCs.

Based on our experiences and other published studies, we wanted to include a mesenchymal marker for the detection of EMT‐like CTCs, frequently present at primary diagnosis and after platinum‐based chemotherapy [29, 52]. In that context, Vim‐positive cells were reported as a useful biomarker in other cancer types [53, 54]. In our patients, these cells occurred at a high frequency; however, they did not necessarily represent mesenchymal sCTCs. Nevertheless, double‐ or triple‐positive CTCs harboring Vim‐positive CTCs (Vim‐/CK‐positive; Vim‐/FRα‐positive; Vim‐/CK‐/FRα‐positive) were detected in individual cases but did not represent reoccurring events in our HGSOC patient cohort. Thus, genetic analysis was not feasible to elucidate their value in the course of the disease.

Finally, the investigation of nuc^high^ cells seems to be promising in future studies. Expressing none of the markers that we postulated to be of relevance in HGSOC, these cells revealed a significant increase in cell count with ongoing treatment, suggesting that either cells evolved toward no antigen expression or nuc^high^ cells were largely unaffected by platinum‐based CTX. Of note, a correlation of ploidy and nuclear staining intensity was not possible due to the small sample size of nuc^high^ sCTCs. However, studies in OC PTs have shown that the nuclear content correlated with a worse prognosis [55, 56]. Finally, these cells harbored a good genomic quality to be further processed. Thus, probably simple FACS sorting with subsequent sequencing of these cells might be a future option to get an impression of tumor cells in HGSOC expanding in the course of the disease.

### 4.2 Genomic features of sCTCs and the PT

In general, the genomic quality of sCTCs, especially at primary diagnosis, was not sufficient for deep sequencing; consequently, our data are more or less observations where we finally can only speculate about the (dis)appearance of clones and their value in the treatment of HGSOC. Apart from nuc^high^ cells, a significant improvement in the overall genomic quality of sCTCs was shown over time, which was not due to a sorting bias, as the quality improvement was also seen across different CTC phenotypes. The improvement in genomic quality may be due to an increased susceptibility of already unstable CTCs to treatment [57], resulting in a more genomic stable population.

Based on our matched sCTC and PT data evaluation from nine patients, different as well as similar CN profiles were detected as known from several types of cancer [58]. Notably, greater similarities were identified between the PT and sCTCs detected at primary diagnosis compared with sCTCs in the follow‐up of the disease. This could be due to a clonal selection of specific genotypes during treatment, as observed in CTCs of patients suffering from colon cancer [59] and currently under further investigation in other cancers [60, 61]. Although the low sequencing depth did not allow further evaluation of clonal associations between cells, it provided evidence for the origin of the sCTCs within each individual.

Only two patients provided PT and matched sCTC data at two different time points, allowing a preliminary patient‐specific evolutionary insight. In this context, sCTCs from multiple sampling time points showed shared PT CNAs in Chr.8, covering the MYC gene. These findings are consistent with research showing that c‐MYC protein levels correlate with platinum resistance [62] and are among the most frequently detected alterations in HGSOC [63]. Other OC studies have modeled tumor evolution using data from PTs and distant metastases [14, 64, 65]. Of note, multi‐omics analysis in 30 primary HGSOC tumors and their matched metastases revealed distinct CNAs in various patient groups. Interestingly, no significant differences were detected when assessing the PT tissue and the matched distant metastases, suggesting that the complex genomic alterations occurred at an early stage of the disease [14].

### 4.3 Genomic features and deeper sequencing of sCTCs

Among all sCTCs, variants in the TP53 gene, believed to be the initiating event in the formation of OC, were frequently detected [15, 16]. Moreover, we confirmed other known HGSOC related variants in our sCTCs, namely *RB1*, *PTEN* (COSM5109), *MYC*, and *ATM* (COSM21825) genes. Although variants in these genes are rare, they have been identified in PTs of HGSOC patients [16], linking our detected sCTCs to the tumor of origin. As the variants were only filtered for a population frequency of < 1%, without controlling for putative somatic variants, these were only anecdotal descriptions of SNVs in sCTCs, and a larger cohort of HGSOC patients is needed to confirm these results.

In addition, the LowPass sequencing data revealed CNA dynamics between different sampling time points. Due to the low sequencing depth, no definitive statement about single‐gene alterations was possible; however, CNAs affecting similar pathways revealed specific features in pretreatment, post‐CTX, and post‐Bevacizumab sCTCs and will be beneficial for further CTC investigations. In particular, because CNAs have been shown to be translated into different gene expression [66].

In pretreatment sCTCs, CNAs were enriched in Chromosomes 2, 7, and 12, which affected DNA repair pathways (Chr.2: MMR pathway, Chr.7: HR pathway). While the HR pathway is known to be largely affected in HGSOC, mainly by mutations of the BRCA genes [16, 67, 68], changes in the MMR pathway are mostly unknown in HGSOC PTs [69], however, if detected, they were related to platinum resistance [70, 71]. Moreover, MMR altered PTs were found to be more likely to respond to PARPi treatment [72]. The CNAs in Chromosome 12 affected multiple pathways (P53‐, RB‐, and RTK/RAS signaling) shown to be involved in HGSOC [14, 16]. While CNAs have been frequently studied in the PTs of HGSOC patients, to the best of our knowledge, CNAs in HGSOC sCTCs are largely unknown. Only one recently published study, investigating sCTCs in five HGSOC patients, revealed heterogeneous sCTC genomes but did not analyze the genomes in more detail [38].

Post‐CTX sCTCs revealed a high frequency of altered segments on Chromosomes 15 and 17. In addition, CNAs affecting the HR and KEGG endometrial cancer pathways were frequently detected in both post‐CTX and post‐Bevacizumab sCTCs. Although PT analysis showed a survival benefit for patients with HR‐deficient tumors [73], our data suggest that the selection of certain CNAs within the HR pathway helped sCTCs to persist under treatment. This is supported by data showing that adaptations within the HR pathway help to avoid apoptosis [74, 75, 76]. Therefore, as several researchers have stated, it may be effective to target further DNA repair mechanisms in HGSOC treatment [77, 78].

Some CNAs were confirmed by Ampli1™ OnkoSeek analysis; however, a high percentage of the sCTCs with genome‐wide CNAs by Ampli1™ LowPass analysis did not reveal single‐gene CNAs. In addition, only few SNV were detected among the investigated sCTCs. This raises the question of whether the genes examined in the Ampli1™ OnkoSeek analysis were an appropriate choice for sCTC investigation in HGSOC patients. Nevertheless, the deep sequencing data revealed an amplification of *CDK4* (Chr.12) in pre‐ and post‐CTX sCTCs from different patients. Further investigations should focus on the *CDK4* gene, especially since CDK4/6 inhibitors have enhanced the treatment of a subgroup of breast cancer patients [79, 80] and recent publications even suggested their use in the treatment of HGSOC [81, 82]. In addition, the newly emerging *ALK* (Chr.2) amplification in sCTCs after chemotherapy should be investigated further, especially since *ALK* was among the deleted genes in sCTCs at primary diagnosis. The *ALK* oncogene is known in the context of non‐small cell lung cancer [83, 84] and colorectal carcinoma [85] and recent investigations of OC xenograft models suggested a sensitization to CTX by targeting downstream targets of *ALK* [86].

Although this study was an exploratory, hypothesis‐generating investigation, we observed that 30% (4/13) of the patients that harbored genetic alterations in their sCTCs showed a progression of the disease and died. However, these preliminary findings require confirmation in larger studies.

### 4.4 Limitations of the study

The study faced key limitations related to marker specificity and genomic analysis. Vim and CK, used in CTC detection, proved unreliable in our HGSOC patients due to high background from leukocytes (Vim) and low genomic quality (CK). While FRα showed promise as an HGSOC‐specific marker, its detection rate was low using the DEPArray™ system. Genomic analysis was further limited by poor DNA quality in many sorted cells, especially pretreatment, restricting sequencing success. Discrepancies between low‐pass and deep sequencing CNA detection complicated data interpretation, and the absence of matched leukocyte data hindered mutation validation. Moreover, a significant limitation was the lack of existing software capable of identifying true shared CNAs between sCTCs and the PT. Since we had no capacity to develop such a tool ourselves, we were restricted to a coarse, manual analysis of the data. Finally, the small cohort and short follow‐up limited conclusions on clinical relevance, emphasizing the need for larger studies and improved protocols focused on high‐quality FRα‐positive and nuc^high^ cells.

## 5 Conclusion

Given the limited sample size, this study should be considered hypothesis‐generating. It does not allow for definitive conclusions regarding the clinical relevance of FRα expression or its impact on disease prognosis or progression. Nevertheless, to the best of our knowledge, this is the first study to assess FRα expression in sCTCs from HGSOC patients. Our follow‐up study on the relevance of sCTCs in the course of HGSOC provided evidence for further investigation of several molecular targets with regard to disease progression. sCTC detection in HGSOC remains challenging and our data highlight the need to establish reliable sCTC identification protocols for further investigation of CTCs. However, these analyses were very cost‐intensive and thus, our study can only be seen as a pilot study to get an impression of the complexity we are facing in HGSOC. Future investigations should focus on marker‐independent CTC detection, for example, analysis of nuc^high^ cells, to increase the analyzable CTC burden in HGSOC patients. CTCs are particularly valuable in understanding disease progression after PT resection, and while this tumor entity lacks universal driver mutations, future experiments should focus on the investigation and interpretation of CNA in HGSOC.

We conclude that OC evolution is likely reflected in the temporal heterogeneity of CTC single cell genomes, providing a potential tool for genomic characterization of evolving resistant phenotypes and disease monitoring approaches.

## Conflict of interest

NHS received research support from Menarini Silicon and Scientific Advisory Borad fees from TZU Cancer Therapeutics. CS has nothing to report. JS has the following stock and other ownership interests, and has received consulting, advisory, travel, or research funding from the listed entities: FAPi Holding (Stock and Other Ownership Interests); Astra Zeneca, Immunoscore, Bayer, Roche, Novartis, Servier, MSD, MSD Sharpe Dome, Bristol‐Myers Squibb/Celgene, Falk Foundation, MCI Deutschland, Eisbach Bio, Abalos Therapeutics, and Boehringer‐Ingelheim. PW discloses the following financial relationships: research funding for their institution from Amgen, AbbVie, AstraZeneca, MSD, GlaxoSmithKline, Novartis, Pfizer, Roche Pharma, Clovis, and Lilly; and honoraria and participation at advisory boards from Amgen, AbbVie, AstraZeneca, MSD, GlaxoSmithKline, Novartis, Pfizer, Roche Pharma, Clovis, TEVA, Eisai, Lilly, Gilead, and Daichii Sankyo. RK has received honoraria for Proctoring, Advisory Board, Presentations, Travel support: Intuitive Surgical, Medtronic, Avatera, CMR Surgical, Active Surgical, Cava Robotics, Distalmotion, Novocure, Medicaroid, GSK, MSD, Astra‐Zeneca, Tesaro. SK‐B served as a consultant for Qiagen. RPLN, STL, JDK, and PB have nothing to report.

## Author contributions

CS and SK‐B contributed to the conceptualization. CS, STL, and RPLN contributed to the methodology. CS contributed to the formal analysis, investigation, data curation, writing—original draft preparation, and visualization. CS, RPLN, NHS, STL, JS, JDK, PB, and SK‐B wrote the manuscript. SK‐B contributed to the supervision. JDK, PW, RK, and SK‐B contributed to the funding acquisition. All authors have read and agreed to the published version of the manuscript.

## Supporting information


**Fig. S1.** (A) Violin plot with Genomic Integrity Index (GII) distribution of 246 FRα‐positive SCs. (B) Violin plot with GII distribution of 187 CK‐positive SCs. (C) Violin plot with GII distribution of 163 cells with no detectable antigen expression (mostly nuc^high^cells). Statistical evaluations were performed with the Kruskal‐Wallis test (ns = non‐significant *P ≤ 0.05 **P ≤ 0.01***P ≤ 0.001****P ≤ 0.0001). before treatment (b.tre.); after CTX (a.CTX); after Bevacizumab (a.Bev.); cytokeratin (CK); folate receptor alpha (FRα); single cells (SNs).

**Fig. S2.** Single gene copy number alterations identified by Ampli1™ OnkoSeek analysis in single circulating tumor cells of high grade serous ovarian cancer patients. Amplification (amp.); deletion (del.); before treatment (b.tre.); after CTX (a.CTX).

**Fig. S3.** Single nucleotide variants in single circulating tumor cells of high grade serous ovarian cancer patients. before treatment (b.tre.); after CTX (a.CTX); after Bevacizumab (a.Bev.).

**Fig. S4.** Single circulating tumor cell gene loci ordered by low copy number alteration detection at the different time points from high grade serous ovarian cancer patients. (A) b.tre., (B) a.CTX and (C) a.Bev. before treatment (b.tre.); after CTX (a.CTX); after Bevacizumab (a.Bev.).

**Table S1.** Clinical characteristics of high grade serous ovarian cancer patients. ^1^FIGO (Fédération Internationale de Gynécology et d'Obstétrique) staging. ^2^Macroscopically complete tumor resection. ^3^Chemotherapy (CTX) regiment: Carbo = Carboplatin, Pac = Paclitaxel, Caelyx = Doxorubicin pegylated liposomal, Bev = Bevacizumab. PT Res = Platinum resistance. ^4^Follow‐up request from 18.04.2023. y = yes; n = no; /= no data available.

## Data Availability

Raw data for this study were generated at Menarini Silicon Biosystems and are available upon request to the corresponding author.
