# Supplementary figures and images for: Phenotypic and genotypic characterization of single circulating tumor cells in the follow‐up of high‐grade serous ovarian cancer
Source: Mol Oncol. 2025 Dec 23;20(6):1535–55. doi: 10.1002/1878-0261.70193 (PMC13238577; doi:10.1002/1878-0261.70193)

**A**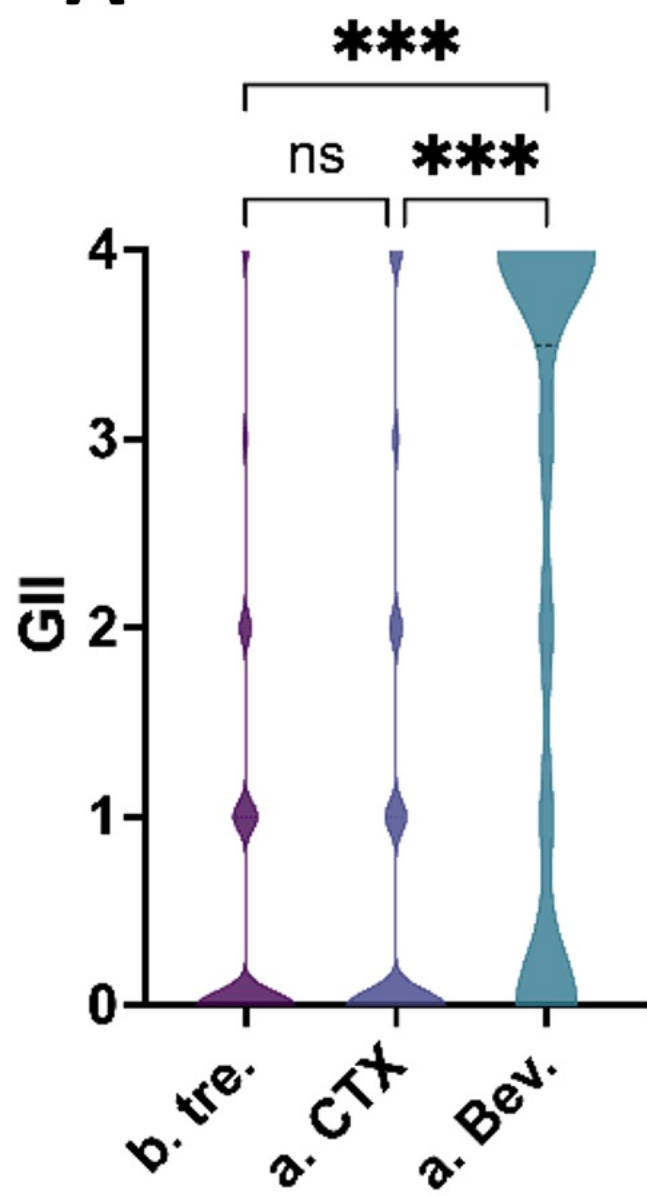**B**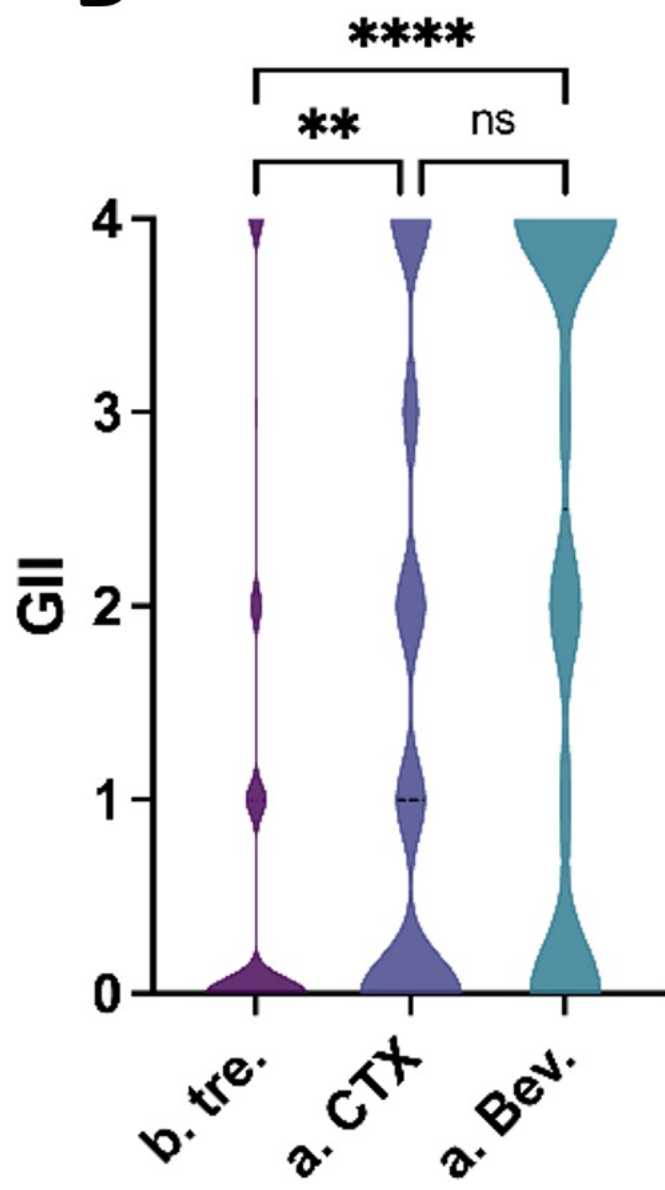**C**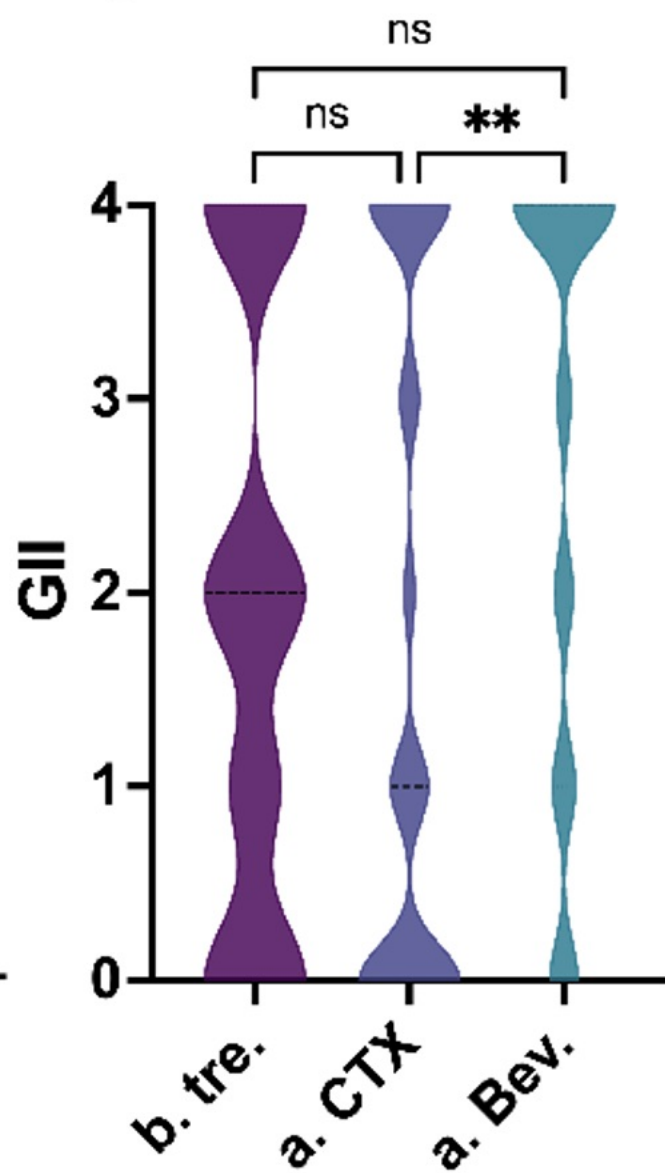

Supplement: Supplementary file 1 — Fig. S1. (A) Violin plot with Genomic Integrity Index (GII) distribution of 246 FRα‐positive SCs. (B) Violin plot with GII distribution of 187 CK‐positive SCs. (C) Violin plot with GII distribution of 163 cells with no detectable antigen expression (mostly nuchighcells). Statistical evaluations were performed with the Kruskal‐Wallis test (ns = non‐significant *P ≤ 0.05 **P ≤ 0.01***P ≤ 0.001****P ≤ 0.0001). before treatment (b.tre.); after CTX (a.CTX); after Bevacizumab (a.Bev.); cytokeratin (CK); folate receptor alpha (FRα); single cells (SNs). [file MOL2-20-1535-s002.pdf]

**A**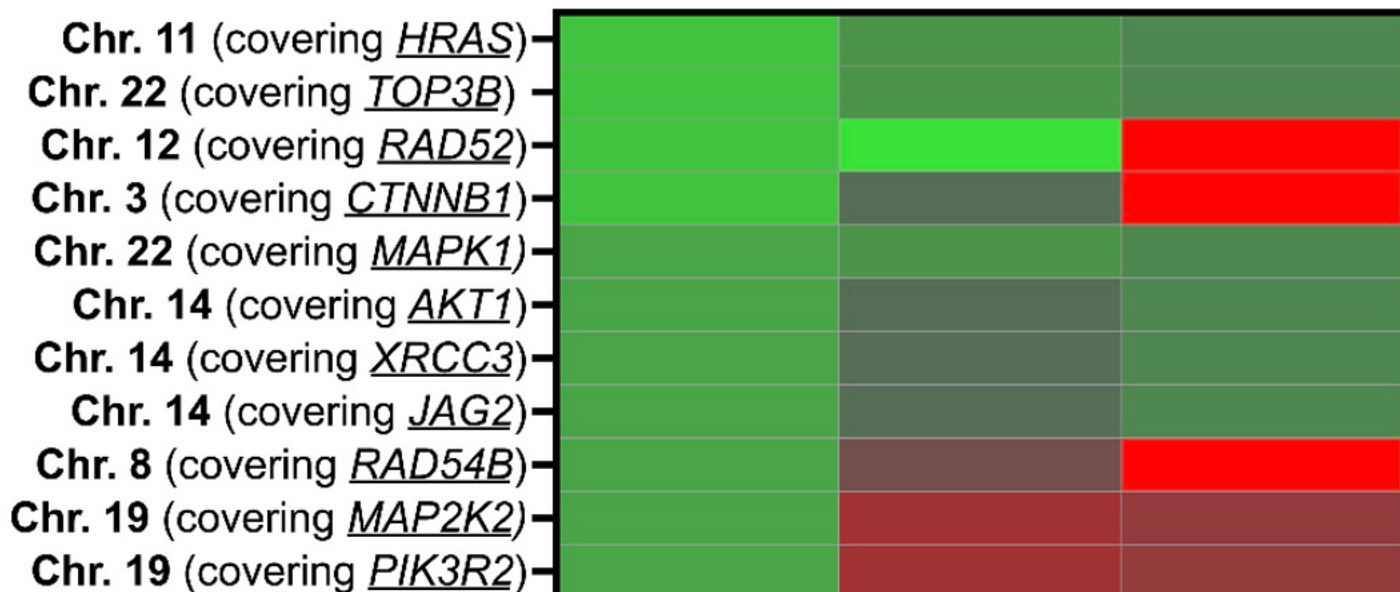**B**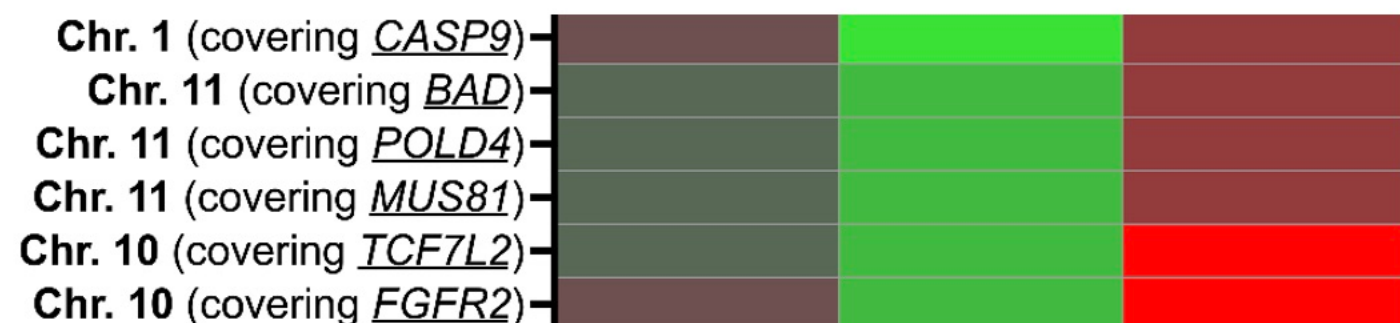**C**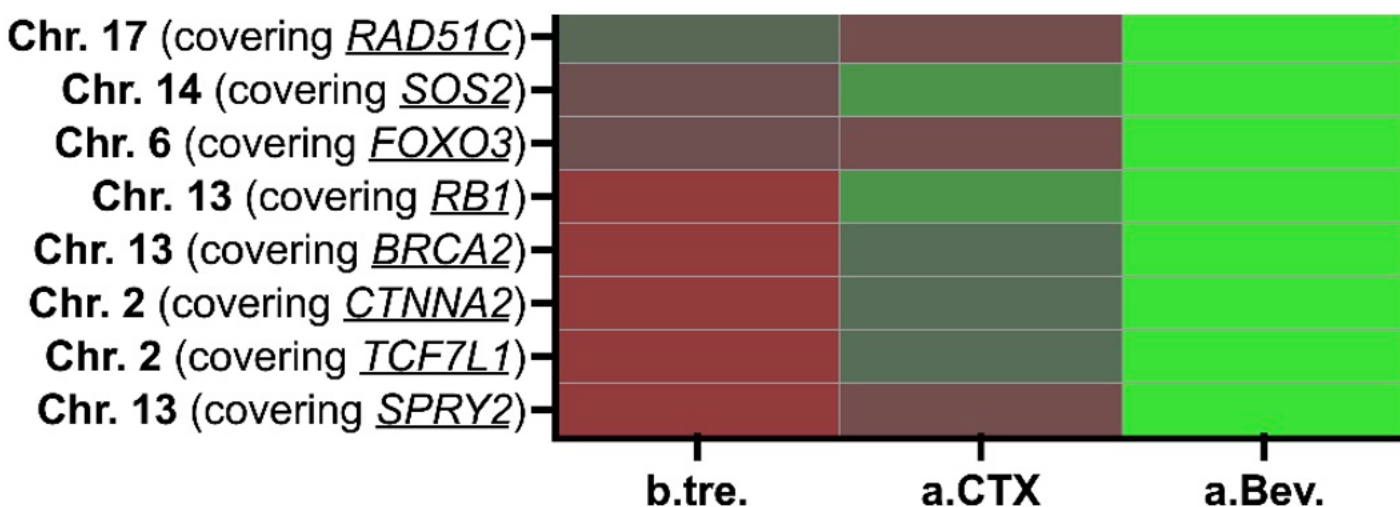

Supplement: Supplementary file 4 — Fig. S4. Single circulating tumor cell gene loci ordered by low copy number alteration detection at the different time points from high grade serous ovarian cancer patients. (A) b.tre., (B) a.CTX and (C) a.Bev. before treatment (b.tre.); after CTX (a.CTX); after Bevacizumab (a.Bev.). [file MOL2-20-1535-s004.pdf]
