# Supplementary material for: Phenotypic and genotypic characterization of single circulating tumor cells in the follow‐up of high‐grade serous ovarian cancer
Source: Mol Oncol. 2025 Dec 23;20(6):1535–55. doi: 10.1002/1878-0261.70193 (PMC13238577; doi:10.1002/1878-0261.70193)
Supplement: Supplementary file 2 — Fig. S2. Single gene copy number alterations identified by Ampli1™ OnkoSeek analysis in single circulating tumor cells of high grade serous ovarian cancer patients. Amplification (amp.); deletion (del.); before treatment (b.tre.); after CTX (a.CTX). [file MOL2-20-1535-s003.pdf]

	**Pat. 10** *100920_46A _F9* **b.tre.**	**Pat. 11** *230920_50A _C6* **b.tre.**	**Pat. 11** *230920_50A _A12* **b.tre.**	**Pat. 11** *230920_50A _B12* **b.tre.**	**Pat. 12** *5_36A _C12* **b.tre.**	**Pat. 37** *134a B6 _2979* **a.CTX**	**Pat. 42** *123a C12 _1346* **a.CTX**
ALK	/	/	/	/	*del.*	*amp.*	*amp.*
CDK4	/	*amp.*	/	/	/	/	*amp.*
BRAF	/	/	*del.*	/	/	/	/
CDK6	/	/	*del.*	/	/	/	/
EGFR	/	/	/	*amp.*	/	/	/
FGFR2	*amp.*	/	/	/	/	/	/
FGFR3	/	/	/	/	*amp.*	/	*del.*
KIT	/	/	/	/	/	/	*del.*
KRAS	/	/	/	/	/	/	*amp.*
MET	/	/	*del.*	/	/	/	/
MYCN	/	/	/	/	*del.*	/	/
PDGFRA	/	/	/	/	/	/	*del.*
PIK3CA	/	/	/	/	/	/	*amp.*
