# Supplementary material for: Phenotypic and genotypic characterization of single circulating tumor cells in the follow‐up of high‐grade serous ovarian cancer
Source: Mol Oncol. 2025 Dec 23;20(6):1535–55. doi: 10.1002/1878-0261.70193 (PMC13238577; doi:10.1002/1878-0261.70193)
Supplement: Supplementary file 3 — Fig. S3. Single nucleotide variants in single circulating tumor cells of high grade serous ovarian cancer patients. before treatment (b.tre.); after CTX (a.CTX); after Bevacizumab (a.Bev.). [file MOL2-20-1535-s005.pdf]

Gene	*Variant*	Allele frequency	*Variant - type*	*Time-point*	*Patient*	*Sample*
TP53	*chr17:7578431: G:A*	20,52	*SNV*	*b. tre.*	**Patient 8**	*120820_43A_F6*
TP53	*chr17:7577072: A:G*	14,88	*SNV*	*b. tre.*	**Patient 8**	*120820_43A_F6*
TP53	*chr17:7579385: T:C*	8,11	*SNV*	*b. tre.*	**Patient 11**	*230920_50A_C6*
TP53	*chr17:7578415: A:G* *	16,79<br>1,36	*SNV*	*b.tre.*<br>*b.tre.*	**Patient 10**<br>**Patient 11**	*100920_46A_F9*<br>*230920_50A_C6*
RB1	*chr13:49033872: T:C*	0,24<br>22,40	*SNV*	*b.tre.*<br>*b.tre.*	**Patient 8**<br>**Patient 12**	*120820_43A_F6*<br>*5_36A_C12*
SMARCB1	*chr22:24133990: C:A*	9,47	*SNV*	*b. tre.*	**Patient 11**	*230920_50A_A12*
PTEN	*chr10:89692818: T:C*	5,49	*SNV*	*b. tre.*	**Patient 11**	*230920_50A_A12*
KIT	*chr4:55593425:G :-*	32,06	*deletion*	*b. tre.*	**Patient 11**	*230920_50A_C6*
PTPN11	*chr12:112888122 :A:G*	29,40	*SNV*	*b. tre.*	**Patient 49**	*129a_C3_3189*
MYC	*chr8:128752879: T:C*	23,68	*SNV*	*a.CTX*	**Patient 37**	*134a_B6_2979*
ATM	*chr11:108119823: T:C*	100,00<br>72,53	*SNV*	*a. CTX*	**Patient 38**	*141a_B3_293*<br>*141a_A12_289*
