# Supplementary material for: Phenotypic and genotypic characterization of single circulating tumor cells in the follow‐up of high‐grade serous ovarian cancer
Source: Mol Oncol. 2025 Dec 23;20(6):1535–55. doi: 10.1002/1878-0261.70193 (PMC13238577; doi:10.1002/1878-0261.70193)
Supplement: Supplementary file 5 — Table S1. Clinical characteristics of high grade serous ovarian cancer patients. 1FIGO (Fédération Internationale de Gynécology et d'Obstétrique) staging. 2Macroscopically complete tumor resection. 3Chemotherapy (CTX) regiment: Carbo = Carboplatin, Pac = Paclitaxel, Caelyx = Doxorubicin pegylated liposomal, Bev = Bevacizumab. PT Res = Platinum resistance. 4Follow‐up request from 18.04.2023. y = yes; n = no; /= no data available. [file MOL2-20-1535-s001.docx]

**Supplement**

Table 1: Clinical characteristics of HGSOC patients. ^1^FIGO staging. ^2^Macroscopically complete tumor resection. ^3^Chemotherapy (CTX) regiment: Carbo= Carboplatin, Pac= Paclitaxel, Caelyx= Doxorubicin pegylated liposomal, Bev=Bevacizumab. PT Res=Platinum resistance. ^4^Follow-up request from 18.04.2023. y=yes; n= no; /= no data available

| **Patient** | **Age at Diagnosis** | | **FIGO^1^** | **BRCA variant** | **R0^2^** | **CTX^3^** | **PARPi** | **Relapse^4^** | **Death^4^** | **PT Res** |
| --- | --- | --- | --- | --- | --- | --- | --- | --- | --- | --- |
| Patient 1 | *58* | *4* | | *n* | *y* | *Carbo+Pac+Bev* | *y* | *y* | *y* | *n* |
| Patient 2 | *76* | *3* | | *y* | *y* | *Carbo+Pac+Bev* | *y* | *y* | *n* | *n* |
| Patient 3 | *69* | *3* | | *n* | *y* | *Carbo+Pac+Bev* | */* | */* | *y* | */* |
| Patient 4 | *60* | *1* | | *y* | *y* | *Carbo+Pac, no Bev* | *n* | */* | *n* | */* |
| Patient 5 | *64* | *3* | | *y* | *n* | *Carbo+Pac, no Bev* | *y* | *y* | *y* | *y* |
| Patient 12 | *78* | *3* | | *n* | *y* | *Carbo+Pac, no Bev* | *y* | */* | *y* | */* |
| Patient 13 | *62* | *2* | | */* | *y* | *Carbo+Pac+Bev* | *n* | *n* | *n* | *n* |
| Patient 6 | *57* | *1* | | *n* | *y* | *Carbo+Pac, no Bev* | *n* | *n* | *n* | *n* |
| Patient 7 | *59* | *3* | | *n* | *n* | *Carbo+Pac+Bev* | *y* | */* | *n* | */* |
| Patient 8 | *72* | *3* | | */* | *y* | *Carbo+Pac+Bev* | *y* | *y* | *y* | *n* |
| Patient 9 | *70* | *4* | | *n* | *y* | *Carbo+Pac+Bev* | *n* | *n* | *n* | *n* |
| Patient 10 | *51* | *4* | | *y* | *y* | *Carbo+Pac+Bev* | *y* | */* | *y* | */* |
| Patient 11 | *60* | *4* | | *n* | *y* | *Carbo+Pac+Bev* | *n/* | *n* | *n* | *n* |
| Patient 14 | *68* | *4* | | *n* | *y* | *Carbo+Pac+Bev* | */* | *y* | *y* | *y* |
| Patient 15 | *44* | *1* | | */* | *y* | *Carbo+Pac, no Bev* | *n* | *n* | *n* | *n* |
| Patient 16 | *59* | *1* | | */* | *y* | *Carbo+Pac, no Bev* | *n* | *n* | *n* | *n* |
| Patient 17 | *54* | *3* | | */* | *y* | *Carbo+Pac+Bev* | *y* | *y* | *n* | *n* |
| Patient 18 | *54* | *3* | | *n* | *n* | *Carbo+Pac+Bev* | *n* | *y* | *y* | *y* |
| Patient 19 | *71* | *3* | | *n* | *y* | *Carbo+Pac+Bev* | *n* | *y* | *y* | *y* |
| Patient 20 | *81* | *3* | | *n* | *y* | *Carbo+Pac+Bev* | *n* | *n* | *n* | *n* |
| Patient 21 | *72* | *3* | | */* | *n* | *n* | *n* | */* | *y* | */* |
| Patient 22 | *51* | *3* | | *n* | *y* | *Carbo+Pac, no Bev* | *y* | *n* | *y* | *n* |
| Patient 23 | *66* | *3* | | */* | *y* | *Carbo+Pac+Bev* | *y* | *y* | *n* | *n* |
| Patient 24 | *76* | *4* | | */* | */* | *Carbo+Pac, no Bev* | *n* | */* | *y* | */* |
| Patient 25 | *65* | *4* | | *n* | *n* | *Carbo+Pac+Bev* | *n* | *y* | *y* | *n* |
| Patient 26 | *72* | *3* | | */* | *n* | *n* | *n* | */* | *y* | */* |
| Patient 27 | *70* | *4* | | */* | *y* | *Carbo+Pac+Bev* | */* | *y* | *n* | */* |
| Patient 28 | *70* | *3* | | */* | *y* | */* | */* | *n* | *n* | */* |
| Patient 29 | *67* | *4* | | */* | *n* | *Carbo+Pac+Bev* | *y* | *n* | *n* | *n* |
| Patient 30 | *65* | *4* | | */* | *y* | *Carbo+Pac+Bev* | *n* | *n* | *n* | *n* |
| Patient 31 | *39* | *1* | | *y* | *y* | *Carboplatin+Caelyx, no Bev* | */* | *n* | *n* | *n* |
| Patient 32 | *59* | *4* | | *n* | *y* | *Carbo+Pac+Bev* | */* | *y* | *n* | *n* |
| Patient 33 | *56* | *4* | | *y* | *n* | */* | */* | */* | */* | */* |
| Patient 34 | *77* | *4* | | *n* | *y* | *Carbo+Pac, no Bev* | *y* | *n* | *n* | *n* |
| Patient 35 | *64* | *3* | | */* | *y* | *Carbo+Pac+Bev* | */* | *n* | *n* | *n* |
| Patient 36 | *63* | *3* | | *n* | *y* | *Carbo+Pac+Bev* | */* | *n* | *n* | *n* |
| Patient 37 | *50* | *3* | | *y* | *y* | *Carbo+Pac+Bev* | *y* | *n* | *n* | *n* |
| Patient 38 | *64* | *3* | | *n* | *y* | *Carbo+Pac, no Bev* | *n* | *n* | *n* | *n* |
| Patient 39 | *67* | *3* | | *n* | *y* | *Carbo+Pac+Bev* | *y* | */* | *n* | */* |
| Patient 40 | *57* | *2* | | */* | *y* | *Carbo+Pac, no Bev* | *n* | *n* | *n* | *n* |
| Patient 41 | *58* | *1* | | */* | *y* | *Carbo+Pac, no Bev* | *n* | *n* | *n* | *n* |
| Patient 42 | *66* | *3* | | *n* | *n* | *Carbo+Pac+Bev* | */* | *y* | *y* | */* |
| Patient 43 | *74* | *3* | | *n* | *n* | *Carbo+Pac+Bev* | *y* | *n* | *n* | *n* |
| Patient 44 | *84* | *3* | | */* | *y* | *Carbo+Bev* | *n* | *n* | *n* | */* |
| Patient 45 | *70* | *3* | | *y* | *y* | *Carbo+Pac+Bev* | *y* | *n* | *n* | *n* |
| Patient 46 | *62* | *3* | | *y* | *y* | *Carbo+Pac+Bev* | *y* | *n* | *n* | *n* |
| Patient 47 | *52* | *3* | | *n* | *y* | *Carbo+Pac+Bev* | *n* | *n* | *n* | *n* |
| Patient 48 | *86* | *3* | | *n* | *n* | *Carbo+Pac+Bev* | *y* | *n* | *n* | *n* |
| Patient 49 | *53* | *3* | | *n* | *y* | *Carbo+Pac+Bev* | *n* | *y* | *n* | *n* |
| Patient 50 | *61* | *4* | | *n* | *y* | *Carbo+Pac* | */* | */* | *n* | */* |
| Patient 51 | *58* | *3* | | *n* | *y* | *Carbo+Pac+Bev* | *n* | */* | *y* | */* |
| Patient 52 | *56* | */* | | */* | */* | *Carbo+Pac, no Bev* | *y* | */* | *y* | */* |
| Patient 53 | *73* | *3* | | *n* | *y* | *Carbo+Pac, no Bev* | *y* | *n* | *n* | *n* |
| Patient 54 | *74* | *1* | | */* | *y* | *Carbo+Pac, no Bev* | *n* | *n* | *n* | *n* |
| Patient 55 | *67* | *4* | | *n* | *n* | *Carbo+Pac, no Bev* | *n* | *y* | *y* | *y* |
